# Crop Wild Relatives as Germplasm Resource for Cultivar Improvement in Mint (*Mentha* L.)

**DOI:** 10.3389/fpls.2020.01217

**Published:** 2020-08-19

**Authors:** Kelly J. Vining, Kim E. Hummer, Nahla V. Bassil, B. Markus Lange, Colin K. Khoury, Dan Carver

**Affiliations:** ^1^ Department of Horticulture, Oregon State University, Corvallis, OR, United States; ^2^ National Clonal Germplasm Repository, USDA-ARS, Corvallis, OR, United States; ^3^ Institute of Biological Chemistry and M.J. Murdock Metabolomics Laboratory, Washington State University, Pullman, WA, United States; ^4^ Decision and Policy Analysis, International Center for Tropical Agriculture (CIAT), Cali, Colombia; ^5^ National Laboratory for Genetic Resources Preservation, Agricultural Research Service, United States Department of Agriculture, Fort Collins, CO, United States; ^6^ Colorado State University, Geospatial Centroid, Fort Collins, CO, United States

**Keywords:** mint, peppermint, spearmint, verticillium wilt, monoterpene

## Abstract

*Mentha* is a strongly scented herb of the *Lamiaceae* (formerly *Labiatae*) and includes about 30 species and hybrid species that are distributed or introduced throughout the globe. These fragrant plants have been selected throughout millennia for use by humans as herbs, spices, and pharmaceutical needs. The distilling of essential oils from mint began in Japan and England but has become a significant industrial product for the US, China, India, and other countries. The US Department of Agriculture (USDA), Agricultural Research Service, National Clonal Germplasm Repository (NCGR) maintains a mint genebank in Corvallis, Oregon. This facility preserves and distributes about 450 clones representing 34 taxa, hybrid species, advanced breeder selections, and F_1_ hybrids. Mint crop wild relatives are included in this unique resource. The majority of mint accessions and hybrids in this collection were initially donated in the 1970s by the A.M. Todd Company, located in Kalamazoo, Michigan. Other representatives of diverse mint taxa and crop wild relatives have since been obtained from collaborators in Australia, New Zealand, Europe, and Vietnam. These mints have been evaluated for cytology, oil components, verticillium wilt resistance, and key morphological characters. Pressed voucher specimens have been prepared for morphological identity verification. An initial set of microsatellite markers has been developed to determine clonal identity and assess genetic diversity. Plant breeders at private and public institutions are using molecular analysis to determine identity and diversity of the USDA mint collection. Evaluation and characterization includes essential oil content, disease resistance, male sterility, and other traits for potential breeding use. These accessions can be a source for parental genes for enhancement efforts to produce hybrids, or for breeding new cultivars for agricultural production. Propagules of *Mentha* are available for distribution to international researchers as stem cuttings, rhizome cuttings, or seed, which can be requested through the GRIN-Global database of the US National Plant Germplasm System, subject to international treaty and quarantine regulations.

## Introduction

The millennia of human effort involved in the domestication of economically important agricultural and horticultural crops is documented and broadly discussed among plant evolutionary biologists (Darwin, 1859; Darlington, 1963; Zohary, 1969; Zohary, 1984; Zohary, 2004). Plant domestication, the genetic modification of a wild form to create an altered plant to meet human needs, has produced many plants incapable of existing in the wild (Doebley et al., 2006). This “domestication syndrome” (Hammer, 1984) involves a combination of traits that are different from those of the wild progenitors. These domesticated plants may have larger fruit or grains, robust growth habits, loss of sexual fertility, loss of bitterness, or synchronous flowering. These plants may not compete successfully in the natural world.

As Zohary (2004) points out, seed propagated agronomic crops, many of which display domestication syndrome, have undergone stabilizing selection to protect fertility. Grain crops have rigid protection for sexual reproduction with streamlined development of flowers, fruits, and seed. Chromosomes behave normally at meiosis and deviants do not survive to reproduce. Chromosomes are balanced with little pollen or seed sterility. In contrast, clonally propagated fruit crops, which also display domestication syndrome, not only tolerate but also promote the reduction of pollen and seed fertility and lower chromosome stability. Parthenocarpy, unequal ploidy levels, aneuploids, and other innovative mass production solutions reduce seed set without reducing fruit production.

At the next level, crops maintained by clonal propagation and grown for their non-reproductive organs have the most drastic disruptions to their flowering and fruiting systems. This group, according to Zohary (2004), demonstrates bizarre chromosomal segregation and unusual ploidy levels. Mint species are an exemplar of this category.

Multiple species of mint have been used for medicinal purposes by humanity from prehistory. From savory herbs produced in monasteries to single-family needs of the kitchen garden, commercial peppermint production for menthol, and essential oil extraction from other mint species, mints represent a significant global economic commodity.

The objectives of this manuscript are to describe the domestication of mint and its uses to humanity. Trait and genotype examples and a summary of the preservation of *Mentha* genetic resources and global genebank operations will be presented. Present breeding improvements and future possibilities considering future genetic analysis will be projected.

## Domestication of Mint

### Taxonomy

*Mentha* is a strongly scented herb genus of the *Lamiaceae* (formerly *Labiatae*) and includes 18 species, 31 subspecies or botanical varieties, and 11 recognized hybrid species (Tucker and Naczi, 2007; GRIN-Global, 2020). The mint family includes diverse additional aromatic genera, such as mountain mint (*Pycnanthemum* L.), lavender (*Lavendula* L.), sage (*Salvia* L.), rosemary (*Rosmarinus* L.), and oregano (*Origanum* L.), which are grown commercially for essential oils that are distilled from leaves and stems. Mint shoots and leaves are used for medicinal and aromatic purposes, such as dried organic extracts, distillates, condiments, and food flavorings. Likewise, *Mentha* includes many commercially valuable species, including peppermint (*Mentha* ×*piperita*), Scotch spearmint (*M*. ×*gracilis*), native spearmint (*M*. *spicata*), American wild mint (*M. canadensis* **)**, and corn mint (*M. arvensis*), which are cultivated in different parts of the world for their culinary and medicinal properties. Plants in this genus are herbaceous, rhizomatous, perennial, and aromatic, with smooth, wide-spreading underground stems, which are square in cross-section. The leaves are arranged in opposite pairs, and the white, pink, or purple flowers are produced in clusters.

The name *Mentha* is derived from Classical Greek mythology, from Minthe, (Minthê), who was a beautiful Cocythian (river nymph) beloved by Hades (Pluto) god of the underworld. Minthe was metamorphosed into dust by Hades’s wife, Demeter (Persephone) (Rosengarten, 1969), but Hades caused the fragrant mint plant to grow from the dust. The etymology of “mint” is from the old English minte (= mint plant), which is derived from Proto-Germanic, through Latin from the Ancient Greek, and is akin to old Norse.

At the base of Mt. Minthe, there was a temple dedicated to Hades and a grove for Demeter (Ovid Met. 10) Near Pylos, Greece, Mt. Minthe is thought to be a location of the origin of triploid sterile spearmint (2*n* = 3*x* = 36), which likely arose from the introgression of the conspecific endemic ancient amphidiploid *Mentha spicata* (2*n* = 4*x* = 48) and the diploid *M. longifolia* (2*n* = 2*x* = 24) (Kokkini, 1991; Lawrence, 1991).

Since the time of Linneaus, more than 3,000 specific epithets have been reported for *Mentha* (Tucker and Naczi, 2007). As a measure of mint species taxonomic diversity, the Global Biodiversity Information Facility (GBIF) network includes more than 740,000 locality data points from 454 *Mentha* taxa with occurrences throughout the world (GBIF, 2020). The plethora of names and occurrences of hybrid and naturalized mints have created confusion in the literature. Wild species of mint hybridize readily, and over evolutionary time, native hybrid-species swarms developed in conspecific regions. Subsequently, minor variances have achieved species rank.

**Figure 1:**
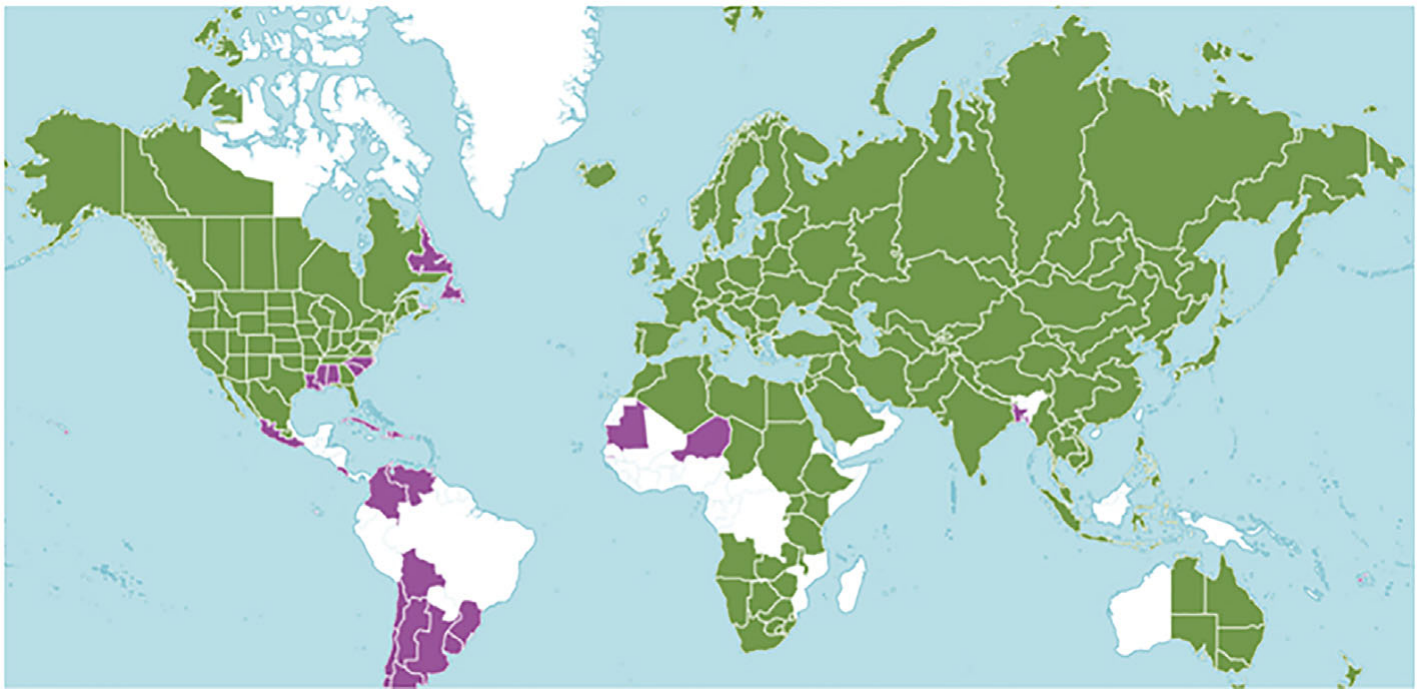
*Mentha* distribution including 39 taxa prepared by Kew Science. The green regions represent native species and purple represents areas of introduction. http://www.plantsoftheworldonline.org/taxon/urn:lsid:ipni.org:names:30016176-2.

The Plants of the World database (http://www.plantsoftheworldonline.org/) (Kew Science, 2020) describes 39 taxa including, 24 species and 15 hybrid species (**Figure 1**). Tucker and Naczi (2007) prepared a more conservative list of the number of mints, synonymizing many to recognize 18 species, 31 subspecies or botanical varieties, 11 hybrid species, and one excluded species. They chose to consider *Mentha cunninghamii* (Benth.) Benth., as *Micromeria cunninghamii* Benth. GRIN-Global (2020) taking a moderate course, includes 20 *Mentha* species.

For this manuscript, we applied the determination by Tucker and others (Tucker and Chambers, 2002; Tucker and Naczi, 2007) for 18 recognized species, with one exception: For this review, we considered *Mentha repens* (J.D. Hook.) Briq. as *M.* *pulegium* L. subsp. *repens*.

### Species Distribution

**Figure 2:**
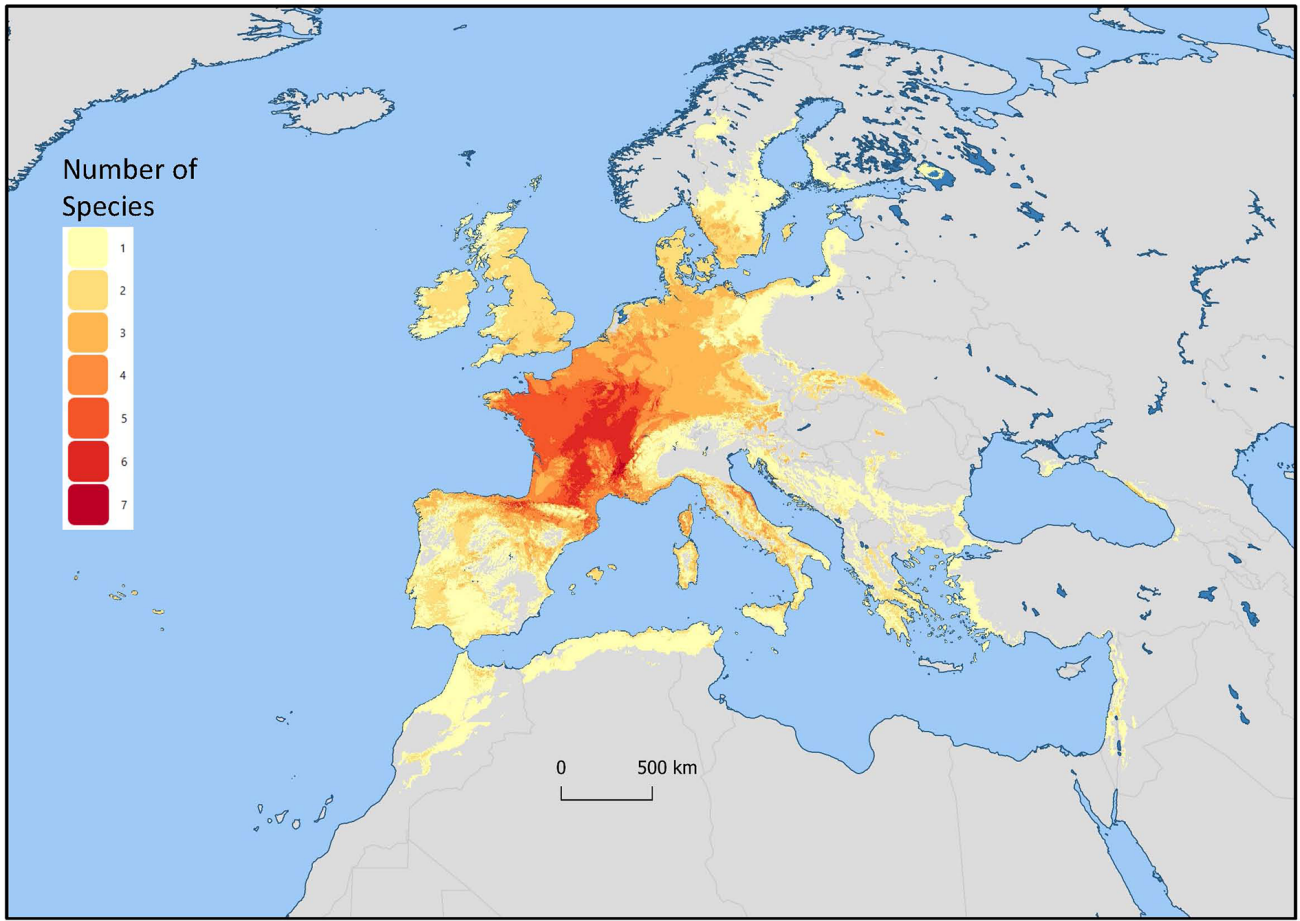
Global richness map for 17 endemic *Mentha* L. species. The colors indicate the number of species in that region.

**Figure 3:**
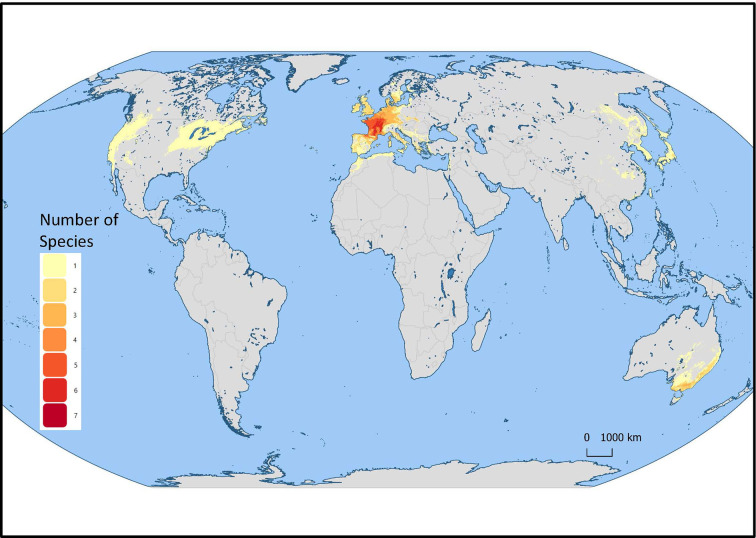
*Mentha* L. European region of the global richness map for 17 endemic European *Mentha* species. The colors indicate the number of species in that region.

*Mentha* L. has a cosmopolitan native range (Kew Science, 2020). The *Mentha* distribution map (Kew Science) included 163 countries, provinces, and regions for native distribution and 43 regions of introduction (**Figure 1**). Considering the tendency for species hybridization within this genus and the successful expansion strategies of mint around the globe, we used a conservative estimation to develop global richness maps. We considered only the taxa accepted by Tucker and Naczi (2007). We prepared global richness maps searching GBIF data for the 17 mint species (**Figures 2** and **3**).

We separately examined world occurrences of point data within GBIF data for each of the 17 species. We filtered out the introduced, naturalized, and cultivated locality data, keeping only natural endemic occurrences. We used climatic and topographic predictors to model likely occurrence for the 17 individual species maps. The data for the separate species maps were merged to produce the global species richness maps (**Figures 2** and **3**). These maps can be used for applied research or conservation planning and investigating the processes that have shaped these patterns.

While the highest diversity of present day species occurs in Western Europe (**Figure 2**), significant endemic mint species occur in Eastern and Western North America, Asia, Southern Australia, and Tasmania (**Figure 3**). In addition to this natural species diversity, hundreds of thousands of data points of introduced, naturalized, and cultivated mint occur throughout five continents (GBIF, 2020). The multitude of global mint occurrences (**Figure 1**) speak to the global success of this genus, starting from Centers of Diversity in Europe, Asia, North America, and Australia and spreading throughout the globe.

The ease of propagation by seeds and clonal propagules, such as rhizomes and cuttings, and the survivability of the plants during harsh and undesirable climactic conditions, allow for spread and diversification. The utilitarian application of mint for human pharmaceutical, food, and cosmetic needs encouraged domestication and cultivation throughout the world and across multiple cultures.

### Prehistory

Throughout human history, medicinal and aromatic plants have been used for flavor enrichment in culinary and medicinal purpose in folk medicine (Sonmezdag et al., 2017). *Mentha* has been of great importance considering its unique aroma and nutritional value (Naghibi et al., 2010). Archeological excavations showed that the usage of lavenders, sage, and mints occurred in prehistoric times, harvested locally from the wild (Nuńez and De Castro, 1992). In western traditions, mints have been cultivated in the dry, mild, and cold districts of Asia, Europe, and North Africa since antiquity (Jamzad et al., 2003). Native peoples in America have documented traditions of uses of mints as cold remedies (Chehalis, Cowlitz), treatments for gastrointestinal ailments (Kiowa), food (Kiowa, chewing leaves), and ceremonial medicine (Navaho) (Vestal and Schultes, 1939; Elmore, 1944; Gunther, 1973).

### Antiquity

Multiple mint species are referenced (Bostok, 1885) throughout Greek and Roman herbals (Aeschylus, Hippocrates, Krataeus, Dioscorides, and Galen; Romans: Cato, Ovid, and Pliny the Elder); Asian medicinal traditions, traditional Chinese medicine, and the Ayurvedic tradition of India.

**Figure 4:**
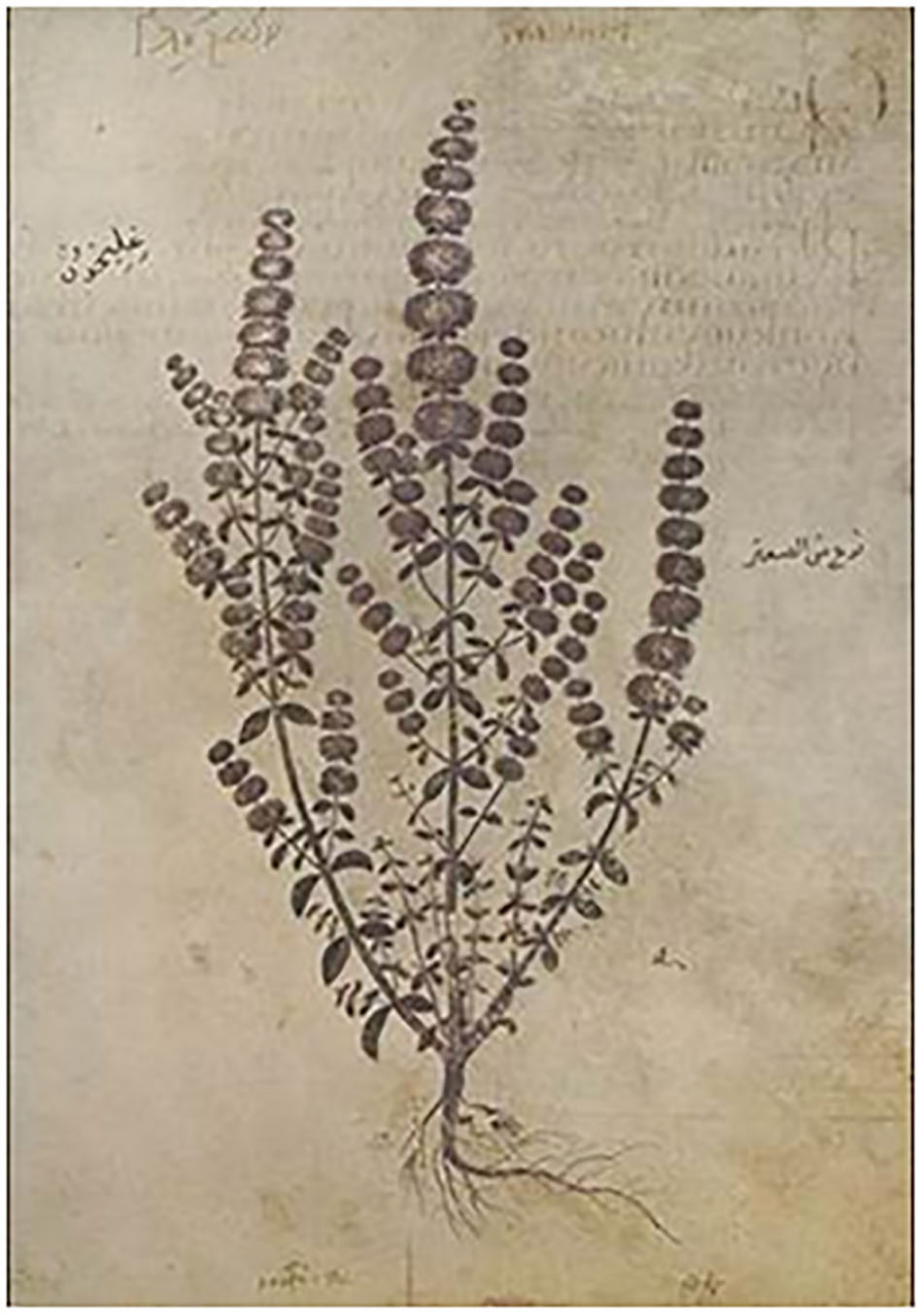
*M. pulegium*, pennyroyal, *Ḡleḵon*, γλήχον Vienna Dioscorides, Juliana Anicia Codex, dating to 512 CE. This illustration is Book III, plate 31. The Arabic writing on the right is translated “type of thyme”. The Arabic on the left is the translation of *Ḡleḵon.* The faded Byzantine Greek in the background is translated by L. Beck (**Table 1**).

**Figure 5:**
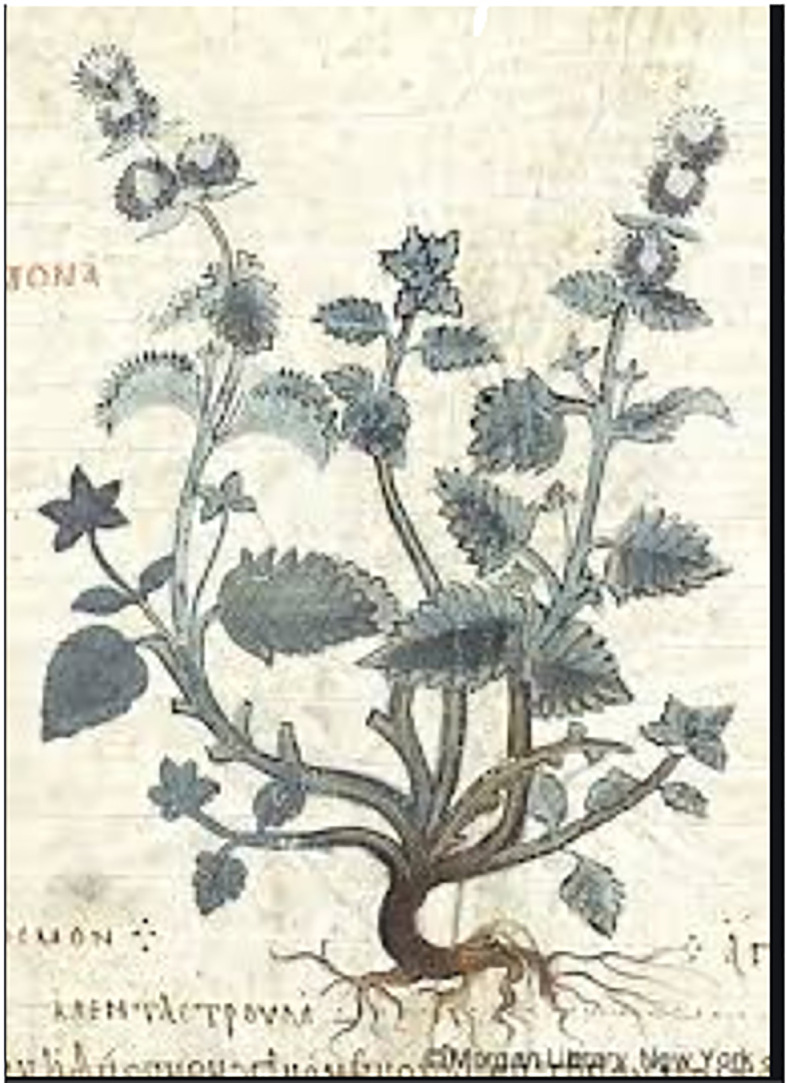
Image of *M. aquatica*, labeled Eduosmos agrios, is from the Pierpont Morgan recession of Dioscorides *De material medica*. This book dates to about 1050 CE, and the plants are alphabetically arranged. Morgan Library New York.

The earliest published mint species images in existence can be found on gr. III fol. 129r and 132r of the Juliana Anicia Codex or Vindobonensis Codex (Janick and Hummer, 2012). The Juliana Anicia Codex is a magnificently illustrated manuscript with written information based on the *Peri Ylis Iatrikis* (De Materia Medica in Latin; Of Medical Matters of Dioscorides). This codex, one of the earliest books, was presented to the imperial Princess Juliana Anicia in Constantinople 512 CE (Collins, 2000). In the 15th century, it was purchased by the Emperor Maximilian for the Imperial Library and was moved to Vienna, where it now resides. The translation of the uses for mints as described in Byzantine Greek written in the background of the images was prepared by Lily Beck (Beck, 2005) and is presented (**Table 1**). These uses described in the Juliana Anicia Codex were reiterated, revised, and interpreted in many recensions of Dioscorides works and in subsequent herbals for the next several millennia (Collins, 2000; **Figures 4** and **5**).

**Table 1:** English translation of reference to species of *Mentha* in *De Materia Medica* of Dioscorides (Beck, 2005).

Book	Translation	
III, 31	[ϒλήχων (βλήχων), *M. pulegium* L. Pennyroyal]	
The pennyroyal: it is a familiar herb, warming, thinning and promoting digestion. When drunk, it draws the menses, afterbirth, and embryos/fetuses. It brings up from the long phlegm when drunk with salt and honey and it helps people with spasms, and with sour wine mixed with water it relieves nausea and gnawing pains of the stomach. With wine, it drives down the bowel dark matter and helps those bitten by wild animals and when applied to the nostrils with vinegar, it revives those who fainted.		
Ground up dry and burned, it also strengths the gums; plastered on with barley groats, it soothes all inflammations; it is suitable to use all by itself on the gouty until the skin surfaces becomes irritated and when used with a cerate, it checks facial eruptions; it also helps patients with spleen disease when plastered on with salt. Its decoction used as a wash stops itching and is suitable in a sitz bath for uterine inflations, indurations, and twistings. Some people call it *blechon* because sheep that taste it when in bloom bleat continuously.		
III, 34		[ύбύοσμον *Mentha* sp. L. Green mint]
The green mint, but some call it *minthe*: it is a well-known little herb having warming, astringent and drying properties; it is for this reason that its juice, when drunk with vinegar, staunches blood, destroys the round intestinal worm, rouses sexual desire, and when two or three little sprays are drunk with the juice of sour pomegranate, stops hiccups, vomiting, and cholera. Applied as a plaster with barley groats, it dissipates abscesses, placed on the forehead, it assuages headaches, and it abates distension and swelling of the breasts.		
With salt, it is a plaster for people bitten by dogs, and its juice with hydromel is suitable for earaches. Used by women as a pessary before sexual intercourse, it causes barrenness, and if rubbed on a rough tongue, it smothes it; it keeps milk from curdling when little sprays are stirred about in it, and it is through and through wholesome and spicy.		
There is also a wild green mint which has thicker leaves, all told it is larger than bergamot mint which has thicker leaves, all told it is larger than bergamont mint, rather foul smelling, and less useful for health purposes.		

Dioscorides mentions about 500 plants (Singer, 1927). About 130 of these were referenced in the Hippocratic collection. Thus, many of these treatments, including peppermint, were utilized in the Greek world for more than four centuries before Dioscorides writings and have survived throughout the times to medicinal applications in the present day.

Mint is mentioned in the bible: Mathew 23:23, “For you tithe mint, dill, and cumin, and have neglected the weightier matters of the law: justice and mercy and faith. It is these you ought to have practiced without neglecting the others” and Luke 11:42, “Woe to you Pharisees, because you give God a tenth of your mint, rue and all other kinds of garden herbs, but you neglect justice and the love of God. You should have practiced the latter without leaving the former undone.” Parry (1925) suggests that these quotations refer to *Mentha longifolia*, long leafed mint, native to North Africa. Robert Gunther (1934) prepared a translation of John Goodyear’s 1655 version of Eduosmos agrios in Dioscorides as: “But ye wilde mint, which ye Romans call *Mentastrum*, is round in the leaves and altogether greater than *Sisymbrium*, but poisonous in smell, and lesse fitting for use in health.”

*Mentha* in the traditional Galenic medicine in the Islamic period (**Table 2**) can be traced to the writings of Dioscorides and other Greek authors.

**Table 2:** Application of mint in Arabic traditional texts. (Iranian encyclopedia online, 2020).

ʿAli b. ʿAbbās Majusi	(date 384/994 CE	Raw mint mixed with vinegar is an effective medicine for treatment of swooning and vomiting.
Ebn Sinā	(d. 428/1037 CE	Mentioned that its sherbet* is useful for curing jaundice.
Jorjāni	(d. 531/1136 CE)	Claimed that it was necessary to eat mint after having cucumber, pumpkin, and the seeds of lettuce.
		Additional references of detailed accounts about mint in traditional Arabic medicine.

*Persian “sherbet”, sekanjabin, a concoction of honey and vinegar flavored with mint extract.

### Egyptian

The *Ebers Papyrus* dating to 1550 BCE and purchased by Georg Ebers in Luxor in 1874, is one of the earliest surviving Egyptian medical papyri. It is kept in the library at the University of Lipzig, Germany. This 110-page scroll is about 20 m long, and the text is scribed in hieratic Egyptian, likely copied from earlier manuscripts. One treatment suggests that peppermint be mixed with flour, incense, wood of the waneb plant, a stag’s horn, sycamore seeds, mason’s plaster, seeds of zart, and water to create a curative paste for headaches supporting later Anglo-Saxon and Greek prescriptions for headache and migraine pain relief (Aboelsoud, 2010; Elansary and Ashmawy, 2013).

In the *Hearst Papyrus*, dated to approximately 2000 BCE, peppermint is recommended as a treatment for rhinitis, where a plaster is applied directly to the nose. In prescription 171, peppermint is mixed with wine and is used to treat what might be edema of the legs. After the mint and wine mixture is consumed, some sort of bloodletting is required, though the physician doesn’t provide much in terms of specificity. Mint is still used to treat edema today, though most modern references deal with topical creams for livestock, particularly cattle (Aboelsoud, 2010).

### European Medieval and Renaissance 1400–1600

The majority of Medieval and Renaissance herbals elaborated the traditions described by ancient Greek and Roman text (Hummer and Janick, 2007). Mint species played a prominent role in many treatments. Adams et al. (2011) compared malaria treatments in Fuchs (1543) and Brunfels (1532), both of whom suggested the use of mints against this disease. Fuchs (1543) also recommended taking pennyroyal (*M. pulegium*) in vinegar against epilepsy. Essential oil extracted from pennyroyal was amongst the pro-convulsive essential oils discussed by Duke (1985), Burkhard et al. (1999), and Riddle and Estes (1992).

Nicholas Culpeper’s work, the *Complete Herbal* (Culpeper, 1652) documents the use of several mint species. Unlike the Egyptian papyri, or Anglo-Saxon Leech books, Culpeper’s work provides in-depth descriptions and growth habits of the plant entries. Culpeper discusses spearmint, which he refers to alternatively as *“heartmint*.*”* The plant is labeled an *“herb of Venus,”* which directly refers to Dioscorides’ *De Materia Medica*, inferring that mint possesses healing, binding, and drying qualities. Much of the entry borrows directly from this text but has some additions. Culpeper includes treatments using mint for a sore and itchy scalp, pain of the ears, venomous bites, headache, indigestion, and flatulence. He also includes prescriptions for the resolution of bad breath and soreness of the gums and palate. Horse or wild mint are then referenced. Due to menthol’s innate analgesic qualities, mint is described to be used against headaches, migraines, and general pain and swelling. Mint is also recommended as a digestive aid. Mint relaxes stomach muscles, allowing food and flatulence to pass more easily.

Mint was employed topically to treat dry, itchy skin, and insect and animal bites and lessen the appearance of scars, bruises and scabs, particularly on the scalp and face. Due to its antimicrobial and antifungal properties, mint was used for millennia to treat fungal infections, such as ringworm, and to treat parasites, such as roundworm. Mint is also a diuretic and, as such, can be used to stimulate urination and possibly even treat hypertension.

## Industrial Production of Mint

Mint was significant to humans from early history, being collected from the wild and brought to the convents, monasteries, and kitchen gardens for direct use. Japan preceded western countries in the cultivation of mint and extraction of menthol from *M. arvensis*, begun before 0 CE (Flueckiger, 1891; Hayden, 1929). Tamba Yasudori (984 CE), in the oldest surviving Japanese medical text, stated that menthol was used in preparation of an aqueous eye medication. Japan began exporting menthol in 1873 (Gildemeister and Hoffman, 1913). In Japan, this oil was extracted from corn mint (*Mentha arvensis* L.).

Production of peppermint for menthol in large areas in the western world did not occur until 1750 in England (Parry, 1925). By 1796, about 40.5 ha (100 acres) of peppermint (*M.* ×*piperita*) were grown near Mitcham, England. The plant material was harvested, bundled, and sent to London for distilling. This early production of peppermint oil yielded 900–1360 kg (2,000–3,000 lb) (Parry, 1925). Over the next 20 years, the production area expanded to 10 townships surrounding Mitcham. By 1940, peppermint cultivation in the original Mitcham area ceased (Parry, 1925).

Peppermint plants were imported from England to the United States with early settlers. By 1812, peppermint was raised commercially near Ashfield, Massachusetts (Lawrence, 1991). Enterprising farmers and distillers, such as Archibald Burnett and Hiram G. Hotchkiss, brought mint westward to New York. By 1830, mint production was sufficient for nine distilleries. These stills also processed spearmint, tansy, wintergreen, spruce, and hemlock oils (Lawrence, 1991). Mint production began to move further west in the US. Both spearmint and peppermint were introduced in New York, Ohio, Michigan, and Indiana. In the western states, mint from Oregon and Washington spread into Idaho, Montana, California, Nevada, and Utah. Lawrence (1991) provides details of early mint production in the US.

In 1853, Albert M. Todd imported ‘Black Mitcham’ roots from England and began production in St. Joseph County, Michigan. ‘White Mitcham,’ the original introduction, had become known as ‘American peppermint.’ Todd found ‘Black Mitcham’ to be superior to ‘White Mitcham’ for production in Michigan. Todd’s production and distillery in Kalamazoo became a major mint producer. By 1915, Todd began several hundred acres of Scotch spearmint (*M.* x *gracilis* Sole), because of its productivity over the native spearmint (*M. spicata*).

The main mint crops produced in the US are peppermint and spearmint, the latter including Scotch and native spearmints. ‘Black Mitcham’ peppermint has been the most widely grown peppermint cultivar since at least the mid-20^th^ century (Lawrence, 1991).

The US does not report peppermint production to the Food and Agriculture Organization of the United Nations (FAOSTAT, 2018). In 2018, the countries reporting the largest peppermint production included Morocco, Argentina, Mexico, Bulgaria, and Spain. Oil distilled in China, India, and Japan is *M. arvensis* var *piperescens* and is not counted in the FAO peppermint database. In 2018, the FAO reported world mint oil production at 106,728 MT.

The annual farm gate value of peppermint production in the US has been between $100 and $150 million, while that of spearmint has ranged between $45 to $50 million during the past decade (NASS, 2020).

Recent US mint oil production was highest in 2011, at about 7 million pounds (3,175 MT) of peppermint oil and 2.2 million pounds (998 MT) of spearmint oil. Since then, US production has dropped to under 6 million pounds (2,721 MT), as production of (-)-menthol from oils of *M. arvensis* has been increasing in China and India. The demand for mint oil by Southeast Asian countries has been driving the expansion of the market in recent years (Liu and Lawrence, 2006).

## Protecting *Mentha* Genetic Resources

The FAO manages a list of world genebanks or botanical gardens and the crops that they preserve. This information is publically available in two global databases: Genesys PGR2 and CATIE. We queried both of these databases on 14 April 2020 and totaled entries for *Mentha* and combined results. This query determined that 65 genebanks in 42 countries as preserving *Mentha* genetic resources. The largest mint collections are held in the US, Ukraine, Germany, Japan, Portugal, and the United Kingdom (**Supplemental Table 1**).

## USDA National Clonal Germplasm Repository at Corvallis, Oregon

The *Mentha* collection of the USDA, NCGR in Corvallis, Oregon, includes representatives of approximately 450 accessions of 13 species and 10 hybrid species plus cultivated types (**Table 3**). This working genebank maintains the primary collections as plants in containers in greenhouse or lath house environments (Hummer et al., 2020). Most of these plants represent diverse wild species collections from 57 countries.

**Table 3:** *Mentha* L. species, distribution, habitat, and number of accessions in the USDA ARS National Clonal Germplasm Repository-Corvallis.

Species	NCGR-Corvallis Accessions	Range	Habitat
*Mentha aquatica L.*	15	Europe except extreme north	wet edges of ponds, lakes, canals
*Mentha aquatica var. citrata*	14	North America and Central Europe	moist fields and roadsides, cultivated
*Mentha arvensis* L.	3	Southern and Western Europe	moist fields and roadsides
	0	Northern and Eastern Europe	moist fields and roadsides
*Mentha australis* R. Br.	2	Australia - except West Australia), Tasmania	near watercourses and waterholes and damp gullies
*Mentha canadensis* L.	43	North America and Eastern Asia	streambankslake shores, moist fields, roadsides. Also cultivated
*Mentha cervina* L.	3	Western Mediterranean, Spain, Portugal, and Mediterranean France	damp land to water overwintering under water in native habitat
*Mentha dahurica* Fisch. ex Benth.	0	Eastern Siberia to Northern China, Hokkaido, Japan	water meadows, shores of rivers and lakes, thickets, wood margins
*Mentha diemenica* Spreng.	1	South Australia to Tasmania and New South Wales, Australia	wet edges of ponds, lakes, canals
*Mentha gattefossei* Maire	5	Grand and Moyen Atlas Mountains, Morocco	edges of dayas, damp pastures
*Mentha grandifora* Benth.	0	Eastern Australia from Inland Queensland to Northern New South Wales	sandy soil
M. hybrids	100	cultivated types	---
*Mentha japonica* (Miq.) Makino.	2	Hokkaido and Honshu, Japan	wet places in lowlands to mountains
*Mentha laxiflora* Benth.	0	Victoria and Southern New South Wales	forests, in gullies, regions above 508 mm rainfall
*Mentha longifolia var. asiatica*	2	Kazakhstan, Kyrgyzstan, Tajikistan, Turkmenistan, Uzbekistan, Russia, China, Afghanistan, India	wet places in lowlands to mountains
Mentha longifolia subsp. longifolia	18	Europe	alpine meadows stream banks above 305 m
Mentha longifolia subsp. dumortieri	0	Belgium	unknown
Mentha longifolia subsp. lavandulacea	0	Spain	unknown
Mentha longifolia subsp. erminea	0	Crete, Central and Southern Greece, Turkey	marshes
Mentha longifolia subsp. cyprica	0	Cyprus Mountains	moist ground by streams and springs
Mentha longifolia subsp. grisella	0	Hungary, Romania, Macedonia, Greece, Asia minor	unknown
Mentha longifolia subsp. diabolina	0	Eastern Europe, Asia	moist areas
Mentha longifolia subsp. mollis	0	Romania, Slovenia, Croatia, Bosnia-Herzegovina, Montenegro, Serbia, Macedonia	unknown
Mentha longifolia subsp. minutiflora	0	Hungary, Macedonia, Crete	unknown
*Mentha longifolia* subsp. *typhoides*	1	Aegean, Northwestern Iran, Northern Iraq, Turkey, Syria, Lebanon, Israel, Egypt	marshy fields by streams and rivers
*Mentha longifolia* subsp. *caucasica*	0	Caucasus	unknown
*Mentha longifolia* subsp. *calliantha*	0	Northwestern Iran, Eastern Anatolia	marshy ground, streamsides
*Mentha longifolia* subsp. *noeana*	0	Western Iran, Iraq, Southeast Anatolia	marshy ground, streamsides
*Mentha longifolia* subsp. *modesta*	0	Asia minor, Iran, Tibet	unknown
*Mentha longifolia* subsp. *royleana*	0	Asia minor, Iran, Afghanistan, Turkestan, Siberia, Tibet	river planes
*Mentha longifolia* subsp. *hymalaiensis*	1	Himalayas, Afghanistan	unknown
*Mentha longifolia* subsp. *syriaca*	0	Syria	wadis
*Mentha longifolia* subsp. *pellita*	0	Syria, Ethiopia	wadis
*Mentha longifolia* subsp. *schimperi*	0	Ethiopia, Sinai Penninsula, Yemin	wadis
*Mentha longifolia* subsp. *capensis*	1	South Africa, Namibia, Zambebwe, Lesotho	in water courses and moist places
*Mentha longifolia* subsp. *polyadenia*	1	South Africa, Lesotho	in water courses and moist places
*Mentha longifolia* subsp. *wissii*		Namibia, South Africa	in water courses and moist places
*Mentha pulegium* var. *pulegium*	9	Southern, Western, and Central Europe, north to Ireland and Central Poland and extending to western and southern Ukraine	wet places, roadsides, pond banks
*Mentha pulegium* var. *micrantha*	0	Southeast Russia, Western Kazakhstan	sinkholes and steppes
*Mentha pulegium* var. *repens*	0	Tasmania, Australia	moist soil
*Mentha requienii* Benth	1	Tyrrhenian Isles (Corsica, Sardinia, Montecristo, Caprera	margins of still and running water, turf, and other damp places often in shade
Mentha satureioides R.BR	0	Australia	native
*Mentha* spp.	6	Australia except in the extreme north	usually loamy soils
*Mentha spicata subsp. spicata*	96	ancient amphidiploid of M. longifolia x M. suaveolens	streambanks, lake shores, moist fields, roadsides; cultivated
*Menhta spicata* subsp *crispata*		cultivated	cultivated
*Mentha spicata* subsp. *condensata*	2	Southern Italy, Sicily, Balkan Penninsula, Agean Region	cultivated
		cultivated	cultivated
*Mentha suaveolens subsp. insularis*	28	Cultivated (western europe from Denmark to eastern Europe and North Africa and the Canaries	along streambaanks, lakeshores, moist field, and roadsides
*Mentha suaveolens subsp. suaveolens*	1	western Mediterranean	islands of the western Mediterranean above 305 m
*Mentha* *suaveolens* subsp. *timja*	0	Morocco	unknown
*Mentha x dalmatica* Tausch	5	Germany, Poland, Switzerland	cultivated
*Mentha x dumetorum* Schult.	2	Europe	cultivated
*Mentha x gracilis* Sole	22	Europe, United States	cultivated, naturalized
*Mentha x piperita* L.	48	Africa, Asia, Australasia, Europe, North and South America	cultivated, naturalized
*Mentha x smithiana* R. A. Graham	3	Europe	native and cultivated
*Mentha x suavis* Guss	1	Africa	native
*Mentha x verticillata* L.	3	Europe	native
*Mentha x villosa* Huds.	6	Europe	native and cultivated
*Mentha x villosa nothovar. alopecuroides* Hull (Briq.)	10	Europe	native and cultivated
Total	455		

Most mints in this collection were originally donated to the USDA from Dr. Merrit J. Murray, breeder at A.M. Todd Company, Kalamazoo, Michigan, dating back to the 1970s. At that time, the Todd Company decided to discontinue their mint breeding program and donated the collection to Dr. Chester Ellsworth Horner, USDA researcher in Corvallis, Oregon. Dr. Al Haunold assumed responsibility upon Dr. Horner’s retirement and subsequently donated the collection to the NCGR in the mid-1980s.

At present, 216 mint accessions at the NCGR are species or advanced breeder selections originally donated from M. J. Murray. Other representatives of diverse mint taxa including wild accessions have since been obtained from breeders and taxonomic collaborators in the US, Australia, New Zealand, Europe, and Vietnam.

Besides maintaining diverse species representatives, the NCGR collection includes advanced breeder lines, cultivar and germplasm releases, and what are referred to as “donor-named selections,” where the NCGR has kept the names provided by donors. In addition, virologists have donated 16 pathogen-positive mints for virus identification studies and certification programs.

## Distribution From the NCGR Mint Gene Bank

The NCGR was dedicated in 1981. Since that time, the NCGR has distributed > 9,700 mints as plants, cuttings, rhizomes, tissue cultures, or seed lots for 1,277 orders. About 31% of the orders were sent to US researchers (including government agencies, universities, and non-profit organizations), 33% to US individuals and private companies, and 36% were shipped to foreign requestors. Additional plant and tissue culture shipments were made for secure remote backup of the collection at the USDA National Laboratory for Genetic Resource Preservation in Ft. Collins, Colorado, the designated base germplasm collection within the US National Plant Germplasm System (NPGS).Some of the most requested cultivars included *M.* × *piperita* ‘Chocolate,’ ‘Pineapple,’ ‘Black Mitcham,’ ‘f. *lavanduliodora*,’ ‘Todd’s Mitcham,’ *M. aquatica* var. *citrata* ‘Eau de Cologne,’ *M*. × *gracilis* ‘Scotch Spearmint,’ and *M. spicata* ‘Kentucky Colonel’ (**Table 4**).

**Table 4:** The 50 most distributed *Mentha* accessions from 1980 through 2020 from the USDA ARS National Clonal Germplasm Repository-Corvallis.

PI number	Species	Cultivar or clonal name	Amount orders shipped
557968	*Mentha* x *piperita*	Chocolate Mint	220
557912	*Mentha suaveolens* subsp*. suaveolens*	Pineapple Mint	113
557922	*Mentha* x *gracilis*	*M* x *gracilis* 10306	104
557952	*Mentha* x *piperita*	*M*. x *piperita* f. lavanduliodora	93
557971	*Mentha x piperita*	Black Mitcham Peppermint	91
557781	*Mentha requienii*	*M. requienii* 10001	79
557939	*Mentha* x *piperita*	White Peppermint	79
277803	*Mentha canadensis*	*M. canadensis* Brazil 701	75
557953	*Mentha x piperita*	*M* x *piperita* 10332	70
557993	*Mentha aquatica* var*. citrata*	Eau de Cologne	70
557974	*Mentha* x *piperita*	*M* x *piperita*	67
557973	*Mentha* x *piperita*	Todd Mitcham Peppermint	63
617475	*Mentha japonica*	*M. japonica* 84232	63
557972	*Mentha* x *piperita*	Murray Mitcham Peppermint	61
617482	*Mentha diemenica*	*M. diemenica*	59
557771	*Mentha pulegium*	*M. pulegium* 10006	58
557935	*Mentha* x *gracilis*	Scotch Spearmint	55
557989	*Mentha aquatica* var*. citrata*	*M*. *aquatica* var. *citrata* 10190	55
557885	*Mentha spicata*	*M. spicata* Oregon	52
557984	*Mentha* x *piperita*	*M.* x *piperita* 10064	52
617476	*Mentha australis*	*M. australis*	52
294099	*Mentha spicata*	*M. spicata* 10085	50
557755	*Mentha longifolia*	*M. longifolia* 10028	49
557758	*Mentha longifolia*	*M. longifolia* 10063	49
557572	*Mentha aquatica*	*M. aquatica* 10176	48
557769	*Mentha longifolia* subsp*. polyadenia*	*M.* *longifolia* subsp. *polyadenia*	47
557770	*Mentha longifolia* subsp*. typhoides*	*M. longifolia* subsp. *typhoides* 10034	47
637833	*Mentha spicata*	Kentucky Colonel	46
557639	*Mentha gattefossei*	*M. gattefossei* 10012	45
557767	*Mentha longifolia subsp. capensis*	*M. longifolia* subsp. capensis	45
557598	*Mentha canadensis*	*M. canadensis* 10247	44
557955	*Mentha* x *piperita*	Murray Mitcham	43
557999	*Mentha suaveolens*	*M. suaveolens* 10084	43
557937	*Mentha* x *piperita*	Mitcham	42
557954	*Mentha* x *piperita*	Todd's Mitcham	42
557913	*Mentha* x *dalmatica*	*M.* x *dalmatica* 10268 Farm #13	41
557597	*Mentha canadensis*	*M. canadensis* Bachmann #2	38
557997	*Mentha aquatica* var*. citrata*	Orange Mint	38
617495	*Mentha canadensis*	*M. canadensis*	38
557634	*Mentha cervina*	*M. cervina* 10013	37
557768	*Mentha longifolia* subsp*. hymalaiensis*	*M. longifolia* subsp. *hymalaiensis*	37
557891	*Mentha suaveolens* subsp*. suaveolens*	*M. suaveolens* 10014 2n rot.	37
557584	*Mentha arvensis*	*M. arvensis* 10260	35
557985	*Mentha aquatica* var. *citrata*	*M. aquatica* var. *citrata* 10185 2n cit.	35
558001	*Mentha* x *smithiana*	*M*. x *smithiana* 10328 En. #5	35
558004	*Mentha* x *verticillata*	*M*. x *verticillata* 10325	35
557911	*Mentha suaveolens subsp. insularis*	*M.* *suaveolens* No. 125	34
557613	*Mentha canadensis*	*M. canadensis* Oregon	33
557918	*Mentha canadensis*	*M. canadensis* 10299	33
617498	*Mentha australis*	*M. australis* 029	33
		Total distributed	2810

Many researchers noted that their purpose for requesting the germplasm was to seek diverse essential oil profiles or to perform genetic analyses. Many universities and companies requested propagules from the entire mint collection for this use. Private company results are unpublished and not publically available. Other scientists were interested in the taxonomy of specific accessions and systematic determinations (Tucker and Chambers, 2002). Others requested germplasm to seek disease resistance (Vining et al., 2005).

The policy of the US NPGS is to distribute plant genetic resources freely for crop improvement. The quarantine importation regulations on international shipment of *Mentha* are relatively minor compared to those for shipment of fruit, nut, and other more restricted horticultural crops. Fewer importation requirements facilitate the ease of transfer of germplasm for foreign requests.

## Germplasm Evaluation and Characterization

The primary objectives of a plant genebank includes the acquisition, maintenance, distribution, evaluation, and characterization of the assigned genetic resources (Chambers and Hummer, 1992). During the past 35 years, staff at the NCGR, working in collaboration with scientists at other institutions, have examined the cytology, essential oil content, disease resistance, and the development and use of molecular markers for identity confirmation and diversity determination. Data from these studies have been added as descriptors to the GRIN-Global database.

## Cytology

Harley and Brighton (1977) defined five sections within the genus *Mentha* (*Mentha* sect. *Audibertia*, sect. *Eriodontes*, sect. *Mentha*, sect. *Preslia*, and sect. *Pulegium*) including 19 species and 13 named hybrids. They also listed chromosome counts for sect. *Mentha* including most of the mint taxa recognized today. Chambers and Hummer (1994) summarized other documentation of chromosome counts and surveyed 73 *Mentha* accessions from the NCGR collection.

Many mint species have a monoploid (base) number of *x* = 12, though diverse species can have monoploid numbers of *x* = 9, *x* = 10, *x* = 18, or *x* = 25. In addition, ploidy series of several species are reported as diploid, tetraploid, hexaploid, octoploid, enneaploid, decaploid, or aneuploid.

Chambers and Hummer (1994) reported that ploidy determinations were obtained by cytological counts of dividing pollen mother cells in flower buds or meristematic cells in root tips. Unusual species counts included *M. requenii* with 2*n* = 2*x* = 18; *M. pulegium* with diploid (2*n* = 2*x* = 20), triploid (2*n* = 3*x* = 30), and tetraploid (2*n* = 4*x* = 40); and *M. japonica* with 2*n* = 2*x* = 50. This survey included chromosome counts for *M. australis* (two accessions*)*, *M. japonica*, *M. diemenica*, and *M. cunninghamii*. For the majority of mint species with *x* = 12, diploid, triploid, tetraploid, hexaploid, octoploid, and decaploid members were observed (Chambers and Hummer, 1994). Vining et al. (2019) recently reported several unusual ploidy counts, finding diploid and triploid clones in *M. suaveolens* and octoploid and enneaploid clones of *M. aquatica* in the NCGR mint collection.

## Essential Oils

The *Lamiaceae*, and particularly *Mentha*, produce aromatic “essential” oils. These lipophilic substances, predominantly terpenes, are produced by capitate or peltate glandular trichomes that occur on the leaf and stem surfaces (Amelunxen, 1964). They can be simply extracted by crushing the leaves and stems or more completely through distillation. The volatile composition is affected by short chain terpenes that constitute the main fraction especially C10 mono- and C15 sesquiterpenes, which overwhelmingly affect the flavor and taste of these species.

**Figure 6:**
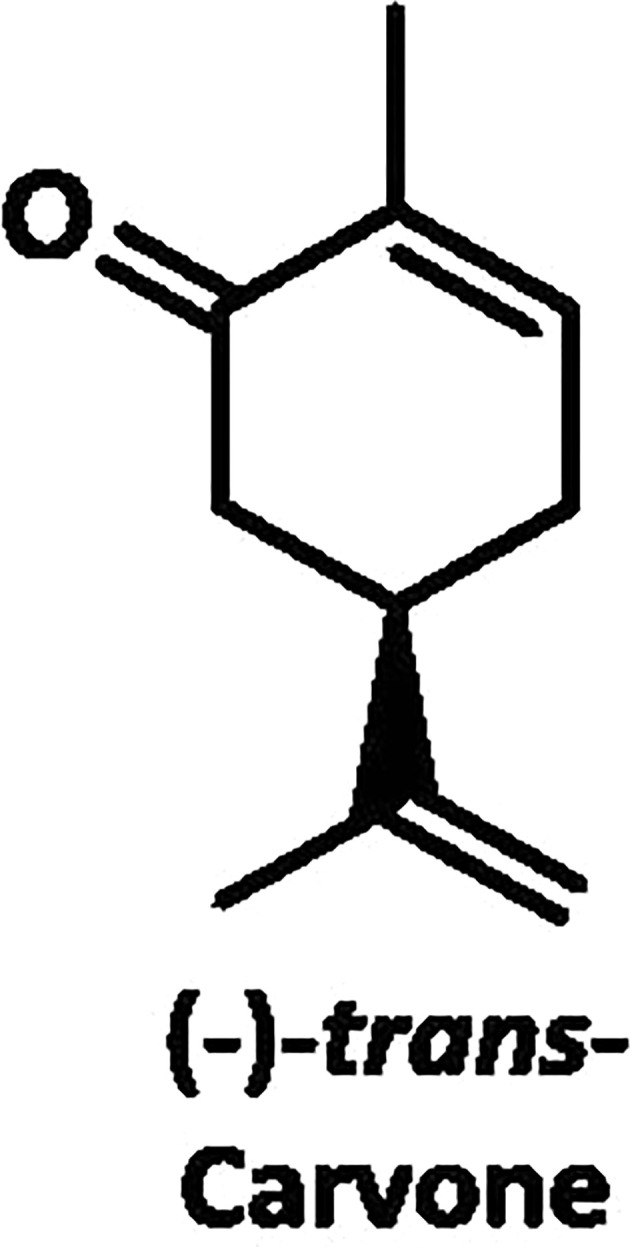
C6-oxygenated p-menthane monoterpene (-)-carvone.

Mints with unique essential oil profiles are valued by the herb, nursery, and pharmaceutical trades. More than 275 accessions of the mint collection of the NCGR were screened by gas chromatography for 37 separate essential oil profiles (Bergstein, 1983). The signature component of native spearmint oil is the C6-oxygenated p-menthane monoterpene (-)-carvone (**Figure 6**). The oil distilled from ‘Black Mitcham’ peppermint contains predominantly C3-oxygenated monoterpenes, with (-)-menthone and (-)-menthol being the most prominent metabolites. Essential oil analyses indicated that about half of the NCGR accessions labeled as *M. aquatica* accumulate (+)-menthofuran as the most abundant monoterpene (with occasionally high levels of (-)-limonene and/or 1,8-cineole as well), while the remainder had variable oil profiles and need to be further investigated (Vining et al., 2019). Oils from *M. suaveolens* accessions are high in either (-)-carvone, piperitenone oxide, or trans-piperitone oxide (Vining et al., 2019). The oil types of *M. longifolia* accessions are quite diverse, with different C6- or C3-oxygenated monoterpenes dominating the profile (Vining et al., 2005).

While the oil composition varies significantly depending on growing location, regional weather, harvest dates, and processing technology (Lawrence, 2006), recent assessments of NCGR accessions and cultivars were performed under controlled conditions to ensure comparability. These data sets can be searched online as descriptors through GRIN-Global (https://npgsweb.ars-grin.gov/gringlobal/method.aspx?id=496325).

## Disease Resistance

Verticillium wilt disease perpetually plagues the mint industry, and therefore breeding for durable wilt resistance is a long-standing goal. Verticillium wilt is a vascular wilt disease caused by the soil-borne fungus *Verticillium dahliae*. ‘Black Mitcham’ peppermint is highly wilt susceptible. However, the cultivar is also a sterile hexaploid, which hinders traditional breeding techniques. Other genetic mutation and recombination techniques have been performed over the years.

An irradiation program, working in collaboration with nuclear facilities in the 1960s, resulted in the release of two new peppermint cultivars with relatively higher wilt resistance: ‘Todd’s Mitcham’ (Todd et al., 1977) and ‘Murray Mitcham’ (Murray and Todd, 1972). In the 1990s–2000s, the Mint Industry Research Council initiated a genetic engineering project with the objective of genetically transforming peppermint cultivars to confer verticillium wilt resistance. However, despite remarkable technical successes in improving oil yield and composition (but not verticillium resistance) (Lange et al., 2011; Lange, 2015), this research was discontinued due to concerns about market acceptance of genetically modified organisms.

## Molecular Analysis

Multi-locus dominant markers such as amplified fragment length polymorphism (AFLP), random amplified polymorphic DNA (RAPD), and intersimple sequence repeat (ISSR) markers have been used with a small number of mint accessions for assessment of genetic diversity and relationships (Khanuja et al., 2000; Gobert et al., 2002; Shasany et al., 2002; Shiran et al., 2004; Vining et al., 2005; Sabboura et al., 2016; Jedrzejczyk and Rewers, 2018; Choupani et al., 2019) and for cultivar identification (Fenwick and Ward, 2001). Species-specific start codon targeted (SCoT) markers were also developed and used to assess genetic diversity in 12 accessions from four mint species (Khanm et al., 2017). Few co-dominant simple sequence repeat (SSR) markers are available for mint (Kumar et al., 2015; Vining et al., 2019). Kumar et al. (2015) identified 54 SSRs from publicly available expressed sequence tag (EST-SSR) sequences of *M.* ×*piperita* that generated clear amplicons in 13 accessions from this species. Three SSRs with long core repeats amplified in representative samples from four species including *M. arvensis*, *M. citrata*, *M. longifolia*, and *M. spicata*, indicating transferability among species.

**Figure 7:**
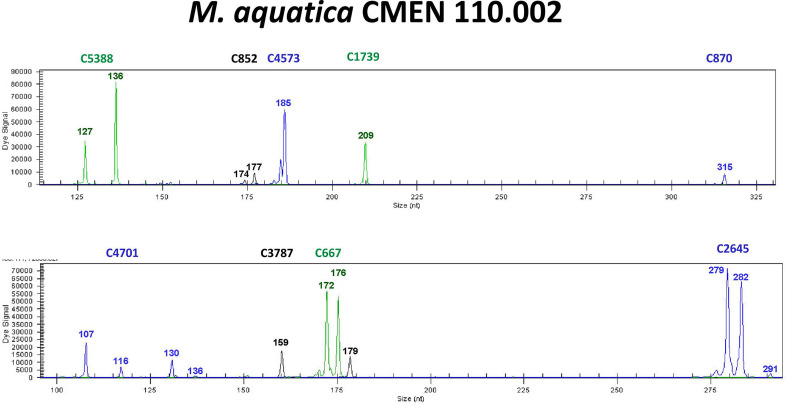
Example of SSR genotyping with *M. aquatica*.

We recently screened these three SSRs in addition to 48 other primer pairs designed from a draft genome assembly of *M. longifolia* CMEN 585, from South Africa (Vining et al., 2017) in an eight-member testing panel (three accessions each of *M. suaveolens* and *M. aquatica* and two accessions of *M. longifolia*) (Vining et al., 2019). Screening the eight-member testing panel with these 51 SSRs identified nine new primer pairs that were polymorphic and appeared easy to score (**Figure 7**). These nine SSRs were thus used in two multiplexes to genotype 49 accessions propagated from the NCGR collection that included 24 accessions of *M. aquatica*, 23 accessions of *M. suaveolens*, and the two *M. longifolia* plants in the testing panel. The nine SSRs developed in this study separated the accessions mostly according to species and identified each accession as unique. Three sets of accessions of *M. aquatica* were closely related and were distinguished by a single allele each: CMEN 116, CMEN 117; CMEN 110, CMEN 111; and CMEN 121, CMEN 122. The consecutive local numbers of each pair of closely related accessions indicate they are in neighboring pots and may suggest plant contamination and will be investigated further.

Single nucleotide polymorphism (SNP) markers have not yet been reported in *Mentha*, and the number of SSRs remains low. More SSRs and new SNPs need to be identified and evaluated across *Mentha* species and are now possible due to the availability of a genome sequence (*M. longifolia*) (Vining et al., 2017), and the low cost of re-sequencing.

## Uses of the Collection in Breeding Efforts

From the mint industry viewpoint, improved oil quality and verticillium wilt disease resistance are two priority traits for new cultivar development. Both traits are multigenic with complex mechanisms. *Mentha* germplasm evaluations have therefore focused on those traits and efforts are ongoing to understand the underlying genetics. Vining et al. (2019) also reported species accessions with undeveloped anthers, resulting in apparent male sterility. This trait could be useful for developing male-sterile cultivars. The USDA NCGR *Mentha* collection is valuable as a source of genetic diversity for mint breeding toward these goals. Several private companies have had, or currently have, mint breeding programs that have requested accessions from the *Mentha* collection. While information about these programs is strictly proprietary, patent documents show cultivar releases for *M. spicata:* (https://patents.google.com/patent/USPP8645P/en; https://patents.google.com/patent/US20120204283A1/en; https://patents.google.com/patent/US20050044600), *M. arvensis* (https://patents.justia.com/patent/PP10935), and an interspecies hybrid: (https://patents.google.com/patent/USPP12030). Besides patents, future cultivars could be released under Plant Variety Protection (PVP), or as germplasm releases through the USDA.

The origin of ‘Black Mitcham’ peppermint, the most widely grown American cultivar in the US and Europe, is unknown, because it resulted from natural hybridization events. However, phylogenetic studies (Tucker et al., 1980; Tucker and Naczi, 2007) suggest that genotypes of *M. longifolia* and *M. suaveolens* are the diploid ancestors of *M. spicata*, which then further hybridized with *M. aquatica* to give rise to *M*. ×*piperita*. Therefore, efforts over the past 50 years have focused on evaluation and crossing of USDA NCGR accessions of *M. longifolia*, *M. suaveolens*, and *M. aquatica*. *M. longifolia* showed the most obvious phenotypic diversity, with variation in growth habit, leaf morphology, and oil type (Vining et al., 2005). *M. suaveolens* and *M. aquatica* showed relatively lower morphological and oil type diversity (Vining et al., 2019).

In *V. dahliae* inoculation tests, both *M. longifolia* and *M. aquatica* showed a range of wilt disease resistance to susceptibility, with a few accessions showing high susceptibility, a few showing high resistance, and most displaying mild to moderate susceptibility levels (Vining et al., 2005; Vining et al., 2019). *M. suaveolens* accessions, in contrast, were highly resistant, with the exception of one moderately susceptible accession (Vining et al., 2019). The contrast in overall wilt disease resistance between *M. suaveolens* and the other two species led to speculation that verticillium wilt disease resistance was genetically linked to a spearmint oil type. However, one carvone-type *M. longifolia* accession, CMEN 584, was among the most highly wilt-susceptible accessions of that species. Segregating F_1_ and F_2_ populations derived from crossing CMEN 584 with wilt-resistant *M. longifolia* accession CMEN 585 also showed a range of wilt resistance to susceptibility, with wilt susceptibility not associated with any particular oil phenotype (Hummer, pers. comm.). It is possible that the higher wilt resistance in *M. suaveolens* results from homozygosity at particular loci or even a different genetic resistance from the other two species.

Transgressive segregation, a phenomenon in which progeny traits differ from those of either parent, is well documented in oil types resulting from *Mentha* crosses (Tucker, 2012). Since both monoterpene profile and verticillium wilt resistance are polygenic traits, transgressive segregation can be expected for verticillium wilt resistance as well. With the advent of the *M. longifolia* reference genome, the genetic underpinnings of these traits are now actively being studied. The complex regulatory mechanisms governing both and verticillium wilt resistance will be a challenge to breeding

In conclusion, mint has a long history of human use and influence and relatively recent (within the past century) increasing attention by mostly private breeding programs. With the advent of genome sequencing, efforts are underway to develop molecular markers for alleles of monoterpene biosynthesis genes and for verticillium wilt disease resistance genes. Going forward, marker assisted selection will increase in importance to mint breeding programs.

## Author Contributions

All authors contributed equally to this manuscript.

## Funding

Funding for the collection, maintenance, distribution, and evaluation of the NCGR Mentha collection was provided by USDA ARS PWA CRIS 2072-21000-049-00D.

## Conflict of Interest

The authors declare that the research was conducted in the absence of any commercial or financial relationships that could be construed as a potential conflict of interest.

The handling editor declared past co-authorship with several of the authors [NB, KV].
