# Supplementary material for: Crop Wild Relatives as Germplasm Resource for Cultivar Improvement in Mint (Mentha L.)
Source: Front Plant Sci. 2020 Aug 19;11:1217. doi: 10.3389/fpls.2020.01217 (PMC7466659; doi:10.3389/fpls.2020.01217)
Supplement: Supplementary file 1 [file DataSheet_1.pdf]

Data source	Institute code	Number of accessions	INSTCODE	ACRONYM	FULL_NAME	TYPE	GENEBANK LONG TERM COLLECTIONS	BOTANICAL GARDEN	GENEBANK MEDIUM TERM COLLECTIONS	GENEBANK SHORT TERM COLLECTIONS	STREET_POB	CITY STATE	ZIP_CODE	PHONE	FAX	EMAIL	URL	LATITUDE	LONGITUDE	ALTITUDE	UPDATED ON	V INSTCODE
Genesys	ALB026	4	ALB026	PGRC	Plant Genetic Resources Center	Governmental	1				Rruga Siri Kodra 132/1	Tirana				agb08document@gmail.com		41.36183	19.76613	null	9/1/2018	ALB026
FAO WIEWS	ALB026	4	ALB026	PGRC	Plant Genetic Resources Center	Governmental	1				Rruga Siri Kodra 132/1	Tirana				agb08document@gmail.com		41.36183	19.76613	null	9/1/2018	ALB026
Genesys	AUT001	13	AUT001	BVAL	AGES Linz - Austrian Agency for Health and Food Safety / Seed Collection	Governmental	1				Wieningerstrasse 8	Linz	A-4020	+43 (0) 50555 - 41231	+43 (0) 50555 - 41119	genetische.ressourcen@ages.at	http://www.genbank.at/	48.26	14.28	null	12/12/2014	AUT001
FAO WIEWS	AUT001	13	AUT001	BVAL	AGES Linz - Austrian Agency for Health and Food Safety / Seed Collection	Governmental	1				Wieningerstrasse 8	Linz	A-4020	+43 (0) 50555 - 41231	+43 (0) 50555 - 41119	genetische.ressourcen@ages.at	http://www.genbank.at/	48.26	14.28	null	12/12/2014	AUT001
Genesys	AUT025	19	AUT025	WIES	Office of the Styrian Regional Government, Department for Plant Health and Special Crops	Governmental	1				Gaisseregg 5	Wies	A-8551	+43 3465 2423-13	+43 3465 2423-30	spezialkulturen-wies@stmk.gv.at	http://www.spezialkulturen.at/offiziell	46.73	15.3	null	12/12/2014	AUT025
FAO WIEWS	AUT025	19	AUT025	WIES	Office of the Styrian Regional Government, Department for Plant Health and Special Crops	Governmental	1				Gaisseregg 5	Wies	A-8551	+43 3465 2423-13	+43 3465 2423-30	spezialkulturen-wies@stmk.gv.at	http://www.spezialkulturen.at/offiziell	46.73	15.3	null	12/12/2014	AUT025
Genesys	AZE004	1	AZE004	IB	Institute of Botany	Governmental					Badamdar Shosse, 40	Baku	AZ1073	(994 12) 439 32 30	(994 12) 439 33 80	botanica@baku.ab.az	http://www.science.az/az/botany/index.htm	40.35637	49.81314	null		AZE004
FAO WIEWS	AZE004	1	AZE004	IB	Institute of Botany	Governmental					Badamdar Shosse, 40	Baku	AZ1073	(994 12) 439 32 30	(994 12) 439 33 80	botanica@baku.ab.az	http://www.science.az/az/botany/index.htm	40.35637	49.81314	null		AZE004
Genesys	AZE014	2	AZE014	ASAU	Azerbaijan State Agrarian University	Governmental				1	262, Ataturk ave.	Ganja	AZ 2000	(+994 22) 56 57 33	(+994 22) 56 24 08	info@adau.edu.az	www.adau.edu.az	40.678523	46.346002	null	4/5/2018	AZE014
FAO WIEWS	AZE014	2	AZE014	ASAU	Azerbaijan State Agrarian University	Governmental				1	262, Ataturk ave.	Ganja	AZ 2000	(+994 22) 56 57 33	(+994 22) 56 24 08	info@adau.edu.az	www.adau.edu.az	40.678523	46.346002	null	4/5/2018	AZE014
Genesys	AZE015	7	AZE015	GRI	Genetic Resources Institute	Governmental	1		1	1	155 Azadlig ave.	Baku	AZ1106	(99412)499129	(99412)499220	akparov@yahoo.com		40.41216	49.83528	null		AZE015
FAO WIEWS	AZE015	7	AZE015	GRI	Genetic Resources Institute	Governmental	1		1	1	155 Azadlig ave.	Baku	AZ1106	(99412)499129	(99412)499220	akparov@yahoo.com		40.41216	49.83528	null		AZE015
Genesys	BEL002	6	BEL002	GxABT	Gembloux agro-biotech, Université de Liège, département des Sciences agronomiques, Phytotechnie tropicale et Horticulture	Governmental	1				Passage des Déportés 2	Gembloux	B - 5030	+32 81 62 21 12	+32 81 62 01 10	gembloux@ulg.ac.be	http://www.gembloux.ulg.be/pc	50.56157	4.69631	null		BEL002
FAO WIEWS	BEL002	6	BEL002	GxABT	Gembloux agro-biotech, Université de Liège, département des Sciences agronomiques, Phytotechnie tropicale et Horticulture	Governmental	1				Passage des Déportés 2	Gembloux	B - 5030	+32 81 62 21 12	+32 81 62 01 10	gembloux@ulg.ac.be	http://www.gembloux.ulg.be/pc	50.56157	4.69631	null		BEL002
FAO WIEWS	BGD003	3	BGD003	BARI	Bangladesh Agricultural Research Institute	Governmental	1	0	1	1	Joydebpur, Gazipur	Gazipur	1701	880-2-9261501-5		dg.bari@bari.gov.bd	www.bari.gov.bd	24.368751	88.661814	null	2/5/2018	BGD003
FAO WIEWS	BGD014	1	BGD014	BFRI	Bangladesh Forest Research Institute (BFRI)	Governmental	1	0	0	0	PO Box 273,	Chittagong	4000	031-681577								
Genesys	BGR001	16	BGR001	IPGR	Institute for Plant Genetic Resources 'K.Malkov'	Governmental	1	1	1	1	Druzba 2	Sadovo, Plovdiv district	4122	+359 32 629026	+359 32 629 026	s_stoyanova@gbg.bg	http://www.genebank.hit.bg/	42.12	24.9	null	3/4/2019	BGR001
FAO WIEWS	BGR001	16	BGR001	IPGR	Institute for Plant Genetic Resources 'K.Malkov'	Governmental	1	1	1	1	Druzba 2	Sadovo, Plovdiv district	4122	+359 32 629026	+359 32 629 026	s_stoyanova@gbg.bg	http://www.genebank.hit.bg/	42.12	24.9	null	3/4/2019	BGR001
Genesys	BRA003	1	BRA003	CENARGEN	Embrapa Recursos Genéticos e Biotecnologia	Governmental	1	0	1	1	Parque Estação Biológica, PqEB, Av. W5 Norte (final)	Brasília-DF	70.770-917	+55 61 3448-4700	+55 61 3340-3624	cenargen.chgeral@embrapa.br	https://www.embrapa.br/recursos-geneticos-e-biotecnologia	-15.45	-47.57	null	5/2/2016	BRA003
Genesys	CHE001	2	CHE001	Agroscope Changins	Agroscope Changins	Governmental	1	0	0	0	Route de Duillier 50, CP 1012	Nyon	CH-1260	(41-58)4604444	(41-22)3621325	info@agroscope.admin.ch	www.agroscope.ch	46.23	6.15	null	10/6/2015	CHE001
FAO WIEWS	CHE001	2	CHE001	Agroscope Changins	Agroscope Changins	Governmental	1	0	0	0	Route de Duillier 50, CP 1012	Nyon	CH-1260	(41-58)4604444	(41-22)3621325	info@agroscope.admin.ch	www.agroscope.ch	46.23	6.15	null	10/6/2015	CHE001
FAO WIEWS	CUB014	2	CUB014	INIFAT	Instituto de Investigaciones Fundamentales en Agricultura Tropical	Governmental					Calle 1 esq. 2, Habana	Santiago de las Vegas-Ciudad	17200	(53) 6834039;6839010;7 6832416; 7 6830093; 7 6830098	(53) 7 6839014	adolforn@inifat.co.cu	www.inifat.co.cu	23.12	-82.25	null	12/12/2014	CUB014
FAO WIEWS	CUB042	1	CUB042	IIHLD	Instituto de Investigaciones Hortícolas Liliana Dimitrova	Governmental	1				Carretera Bejucal-Quivicán, Km 33 1/2. Quivicán.	Quivicán, La Habana	33 500	(53) 47 682600;681603-05	(53) 47 682601	direccion@liliana.co.cu		22.23	-82.23	null	12/12/2014	CUB042
Genesys	CZE061	25	CZE061	CRI	Gene bank - Vegetables and special crops	Governmental	1		1		Slechtitelu, 11	Olomouc - Holice	783 71	+420-585 208966		VURV@genobanka.cz	http://genbank.vurv.cz/genetic/resources/	49.75	17.25	null		CZE061
FAO WIEWS	CZE061	25	CZE061	CRI	Gene bank - Vegetables and special crops	Governmental	1		1		Slechtitelu, 11	Olomouc - Holice	783 71	+420-585 208966		VURV@genobanka.cz	http://genbank.vurv.cz/genetic/resources/	49.75	17.25	null		CZE061
Genesys	CZE122	4	CZE122	CRI	Gene bank	Governmental	1	0	1	0	Drnovska 507/73	Prague 6 - Ruzyne	161 06	+420 233 022 497	+420 233 311 591	cropscience@vurv.cz	http://www.vurv.cz	50.086535	14.302742	null	5/1/2018	CZE122
FAO WIEWS	CZE122	4	CZE122	CRI	Gene bank	Governmental	1	0	1	0	Drnovska 507/73	Prague 6 - Ruzyne	161 06	+420 233 022 497	+420 233 311 591	cropscience@vurv.cz	http://www.vurv.cz	50.086535	14.302742	null	5/1/2018	CZE122
Genesys	DEU022	9	DEU022	BGBM	Botanical Garden Berlin-Dahlem	Governmental	1	1			Koenigin-Luise-Strasse 6-8	Berlin	14191	030/83850-100	030/83006-186	zebgbm@zedat.fu-berlin.de	http://www.bgbm.fu-berlin.de/bgbm	52.45617	13.30634	null		DEU022
FAO WIEWS	DEU022	9	DEU022	BGBM	Botanical Garden Berlin-Dahlem	Governmental	1	1			Koenigin-Luise-Strasse 6-8	Berlin	14191	030/83850-100	030/83006-186	zebgbm@zedat.fu-berlin.de	http://www.bgbm.fu-berlin.de/bgbm	52.45617	13.30634	null		DEU022
Genesys	DEU146	225	DEU146	IPK	Genebank, Leibniz Institute of Plant Genetics and Crop Plant Research	Governmental	1				Corrensstrasse 3	Gatersleben	6466	+49 (0)39482 5220	+49 (0)39482 5500	graner@ipk-gatersleben.de	http://www.ipk-gatersleben.de	51.82599	11.27803	null		DEU146
FAO WIEWS	DEU146	225	DEU146	IPK	Genebank, Leibniz Institute of Plant Genetics and Crop Plant Research	Governmental	1				Corrensstrasse 3	Gatersleben	6466	+49 (0)39482 5220	+49 (0)39482 5500	graner@ipk-gatersleben.de	http://www.ipk-gatersleben.de	51.82599	11.27803	null		DEU146
Genesys	DEU502	10	DEU502		Botanical Garden of the University of Osnabrück	Governmental	1	1			Albrechtstrasse 29	Osnabrück	49076	0541/969-2704	0541/969-2724		http://www.bogos.uos.de/	52.28053	8.02756	null		DEU502
FAO WIEWS	DEU502	11	DEU502		Botanical Garden of the University of Osnabrück	Governmental	1	1			Albrechtstrasse 29	Osnabrück	49076	0541/969-2704	0541/969-2724		http://www.bogos.uos.de/	52.28053	8.02756	null		DEU502
Genesys	DEU515	17	DEU515		Botanischer Versuchs- und Lehrgarten der Universitaet Regensburg	Governmental	1				Universitaetsstrasse 31	Regensburg	93053	0941/9433-295	0941/9433-106		http://www.biologie.uni-regensburg.de/ZentraleEinrichtungen/BotanischerGarten/index.html	48.99326	12.09101	null		DEU515
FAO WIEWS	DEU515	17	DEU515		Botanischer Versuchs- und Lehrgarten der Universitaet Regensburg	Governmental	1				Universitaetsstrasse 31	Regensburg	93053	0941/9433-295	0941/9433-106		http://www.biologie.uni-regensburg.de/ZentraleEinrichtungen/BotanischerGarten/index.html	48.99326	12.09101	null		DEU515
Genesys	DEU626	12	DEU626		Pädagogische Hochschule Karlsruhe		1				Bismarckstraße 10	Karlsruhe	76133	+49 721 925 3	+49 721 925 4000	poststelle(at)ph-karlsruhe.de	http://www.ph-karlsruhe.de/anschrift/	49.01324	8.39326	null		DEU626
FAO WIEWS	DEU626	12	DEU626		Pädagogische Hochschule Karlsruhe		1				Bismarckstraße 10	Karlsruhe	76133	+49 721 925 3	+49 721 925 4000	poststelle(at)ph-karlsruhe.de	http://www.ph-karlsruhe.de/anschrift/	49.01324	8.39326	null		DEU626
FAO WIEWS	ECU023	64	ECU023	DENAREF	Departamento Nacional de Recursos Fitogenéticos	Governmental	1	1	1	1	Panamericana Sur km1 POB17-01-340	Quito, Pichincha		(5932)2693359	(5932)2693359	denaref@ecnet.ec	http://www.denaref.org	-0.14	-78.25	null		ECU023
FAO WIEWS	ECU331	1	ECU331	G.E. Socavón	Granja experimental Socavón	Governmental	1	0	1	0		San Cristobal				yolanda.valverde@iniap.gob.ec	www.iniap.goc.ec	-0.9091	-89.551	null		ECU331
FAO WIEWS	ESP003	6	ESP003	UPM-BGV	Comunidad de Madrid. Universidad Politécnica de Madrid. Escuela Técnica Superior de Ingenieros Agrónomos. Banco de Germoplasma	Governmental	1				Ciudad Universitaria s/n	Madrid	28040	-913365627	-913365622	bgv.agromomos@upm.es	http://www.bancodegermoplasma.upm.es/	40.27	-3.43	null	12/12/2014	ESP003
Genesys	ESP010	2	ESP010	SIAEX	Junta de Extremadura. Dirección General de Ciencia y Tecnología. Centro de Investigación Agraria Finca La Orden - Valdesequera.	Governmental			1		A-V km 372. Apdo. 22	Guadajira-Lobón. Badajoz	6080	-924013966	-924013967	francisco.gonzalezlopez@juntadeextremadura.net	http://centrodeinvestigacionlaorden.es	38.84	-6.67	null	12/12/2014	ESP010
FAO WIEWS	ESP010	2	ESP010	SIAEX	Junta de Extremadura. Dirección General de Ciencia y Tecnología. Centro de Investigación Agraria Finca La Orden - Valdesequera.	Governmental			1		A-V km 372. Apdo. 22	Guadajira-Lobón. Badajoz	6080	-924013966	-924013967	francisco.gonzalezlopez@juntadeextremadura.net	http://centrodeinvestigacionlaorden.es	38.84	-6.67	null	12/12/2014	ESP010
Genesys	ESP027	4	ESP027	CITA-HOR	Gobierno de Aragón. Centro de Investigación y Tecnología Agroalimentaria. Banco de Germoplasma de Hortícolas	Governmental	1				Avda. Montanana 177. Apdo. 727	Montañana. Zaragoza	50080	-976716328	-976716301	cmallor@aragon	http://www.cita-aragon.es	41.41	-0.48	null	12/12/2014	ESP027
FAO WIEWS	ESP027	4	ESP027	CITA-HOR	Gobierno de Aragón. Centro de Investigación y Tecnología Agroalimentaria. Banco de Germoplasma de Hortícolas	Governmental	1				Avda. Montanana 177. Apdo. 727	Montañana. Zaragoza	50080	-976716328	-976716301	cmallor@aragon	http://www.cita-aragon.es	41.41	-0.48	null	12/12/2014	ESP027
Genesys	ESP109	8	ESP109	ITACYL	Junta de Castilla y León. Instituto Tecnológico Agrario de Castilla y León. Centro de Investigación de Zamadueñas	Governmental			1		Ctra. de Burgos km 119. Apdo. 172	Valladolid	47080	-983414397	-983414746	camsalco@itacyl.es	http://www.itacyl.es	41.39	-4.43	null	12/12/2014	ESP109
FAO WIEWS	ESP109	8	ESP109	ITACYL	Junta de Castilla y León. Instituto Tecnológico Agrario de Castilla y León. Centro de Investigación de Zamadueñas	Governmental			1		Ctra. de Burgos km 119. Apdo. 172	Valladolid	47080	-983414397	-983414746	camsalco@itacyl.es	http://www.itacyl.es	41.39	-4.43	null	12/12/2014	ESP109
Genesys	ESP218	3	ESP218	JBCLM	Jardín Botánico de Castilla- La Mancha	Governmental		1			Avenida de La Mancha 1	Albacete	2071	+00 34 967 238820	+ 00 34 967 599 238	info@jardinbotanico-clm.com	http://www.jardinbotanico-clm.com/	39	1.52	null	12/12/2014	ESP218
FAO WIEWS	ESP218	3	ESP218	JBCLM	Jardín Botánico de Castilla- La Mancha	Governmental		1			Avenida de La Mancha 1	Albacete	2071	+00 34 967 238820	+ 00 34 967 599 238	info@jardinbotanico-clm.com	http://www.jardinbotanico-clm.com/	39	1.52	null	12/12/2014	ESP218
FAO WIEWS	FIN038	6	FIN038	KAO Seppälä	Kainuu Region Vocational College	Governmental	1	0	1	0	Opintie 3	Kajaani	FI-87100	+358 8 616 51			www.kao.fi	64.2158	26.7201	null		FIN038
Genesys	GBR004	66	GBR004	RBG	Millennium Seed Bank Project, Seed Conservation Department, Royal Botanic Gardens, Kew, Wakehurst Place	Governmental	1	1			Wakehurst Place, Ardingly	Ardingly, near Haywards Heath,West Sussex	RH17 6TN	+44(0) 1444 894100	+44(0) 1444 894110	Seedbank@rbgkew.org.uk	http://www.rbgkew.org.uk/msbp/	51.07	-0.08	null	12/12/2014	GBR004
FAO WIEWS	GBR004	48	GBR004	RBG	Millennium Seed Bank Project, Seed Conservation Department, Royal Botanic Gardens, Kew, Wakehurst Place	Governmental	1	1			Wakehurst Place, Ardingly	Ardingly, near Haywards Heath,West Sussex	RH17 6TN	+44(0) 1444 894100	+44(0) 1444 894110	Seedbank@rbgkew.org.uk	http://www.rbgkew.org.uk/msbp/	51.07	-0.08	null	12/12/2014	GBR004
FAO WIEWS	GUY021	1	GUY021	NAREI	National Agricultural Research and Extension Institute	Governmental	1	0	0	1	Agriculture Road	Mon Repos, East Coast Demerara	414221H	(592) 220-2249	(592 220-4481	insap@guyana.net.gy	http://agriculture.gov.gy/	6.798	-58.055	null	2/5/2018	GUY021
Genesys	HRV041	45	HRV041	FAZ	Faculty of Agriculture, University of Zagreb	Governmental	0	0	1	0	Svetošimunska cesta 25	Zagreb	HR-10000	385 1 239 3777	385 1 231 5300	dekanat@agr.hr	http://www.agr.unizg.hr	45.8276	16.0295	null		HRV041
FAO WIEWS	HRV041	41	HRV041	FAZ	Faculty of Agriculture, University of Zagreb	Governmental	0	0	1	0	Svetošimunska cesta 25	Zagreb	HR-10000	385 1 239 3777	385 1 231 5300	dekanat@agr.hr	http://www.agr.unizg.hr	45.8276	16.0295	null		HRV041
Genesys	HUN003	7	HUN003	RCA	Institute for Agrobotany	Governmental	1				Kulsomezo 15	Tápiószele	H-2766	(36-53)380070/1	(3653)380072		http://www.rcat.hu	47.25	19.54	null	12/12/2014	HUN003
FAO WIEWS	HUN003	7	HUN003	RCA	Institute for Agrobotany	Governmental	1				Kulsomezo 15	Tápiószele	H-2766	(36-53)380070/1	(3653)380072		http://www.rcat.hu	47.25	19.54	null	12/12/2014	HUN003
FAO WIEWS	IND001	7	IND001	NBPGR	National Bureau of Plant Genetic Resources	Governmental	1		1	1	Pusa Campus	New Delhi	110012	91-11-25843697	91-11-25842495	director@nbpgr.ernet.in	http://www.nbpgr.ernet.in	28.32	77.12	null	12/12/2014	IND001
Genesys	ISR002	45	ISR002	IGB	Israel Gene Bank for Agricultural Crops, Agricultural Research Organisation, Volcani Center	Governmental	1				Volcani Center, P.O. Box 6	Bet Dagan	50-250	(972-3)9683896	(972-3)9669642			32	34.83	null	12/12/2014	ISR002
FAO WIEWS	ISR002	45	ISR002	IGB	Israel Gene Bank for Agricultural Crops, Agricultural Research Organisation, Volcani Center	Governmental	1				Volcani Center, P.O. Box 6	Bet Dagan	50-250	(972-3)9683896	(972-3)9669642			32	34.83	null	12/12/2014	ISR002
Genesys	ITA389	2	ITA389	CREA-FL-TN	CREA-Centro di Ricerca Foreste e Legno, sede di Trento	Governmental	1	0	0	0	Piazza Nicolini, 6	Trento loc. Villazzano	38100	0039 0461 381111	0039-0461-381138	fl@crea.gov.it	http://sito.entecra.it/portale/cra_dati_istituto.php?id=217&lingua=IT&access_flag=0	46.02	11.08	null		ITA389
FAO WIEWS	ITA389	2	ITA389	CREA-FL-TN	CREA-Centro di Ricerca Foreste e Legno, sede di Trento	Governmental	1	0	0	0	Piazza Nicolini, 6	Trento loc. Villazzano	38100	0039 0461 381111	0039-0461-381138	fl@crea.gov.it	http://sito.entecra.it/portale/cra_dati_istituto.php?id=217&lingua=IT&access_flag=0	46.02	11.08	null		ITA389
Genesys	JOR015	2	JOR015		Agricultural Research Service													null	null	null		JOR015
FAO WIEWS	JOR105	2	JOR105	NARC	National Agricultural Research Center	Governmental	1	0	1	1	P.O. Box 639	Baqa'	19381	++ 962 64725071	++ 962 64726099	director@narc.gov.jo	http://www.narc.gov.jo/	32.079864	35.842311	null	5/3/2019	JOR105
FAO WIEWS	JPN183	159	JPN183	NARO	NARO Genebank	Governmental	1	0	0	0	2-1-2 Kannondai	Tsukuba-shi, Ibaraki-ken	305-8602	-7661			http://www.gene.affrc.go.jp/about_en.php	36.03005	140.09793	null	9/1/2018	JPN183
Genesys	LBN002	3	LBN002	ICARDA	International Centre for Agricultural Research in Dry Areas	CGIAR					P.O. Box 114/5055	Beirut		22509				null	null	null	12/12/2014	LBN002
FAO WIEWS	LBN020	4	LBN020	LARI	Lebanese Agricultural Research Institute	Governmental	0	0	0	1	Tal Amara	Zahle		00 961 8 90 00 37/ 47 / 57	00 961 8 90 00 77	lari@lari.gov.lb	www.lari.gov.lb	33.861333	35.990583	null	1/12/2017	LBN020
FAO WIEWS	MAR088	3	MAR088	INRA CRRAS	Centre Régional de la Recherche Agronomique de Settat	Governmental	1		1	1	Route Ain Nzagh B.P. 589	Settat	26000	+212 (0) 23 72 93 00	+212 (0) 23 40 32 09	arido@inra.org.ma	http://www.inra.org.ma/centres/cras/cras.htm	33	-7.62	null	12/12/2014	MAR088
Genesys	MDA011	29	MDA011	IGPPP	Institute of Genetics, Physiology and Plant Protection	Governmental	0	0	0	0	20, Padurii str.	Chisinau	MD-2002	(+373 22) 77 04 47	(+373 22) 55 6180	asm_igfpp@yahoo.com	http://igfpp.asm.md/	46.98	28.89	null	11/7/2017	MDA011
FAO WIEWS	MDA011	29	MDA011	IGPPP	Institute of Genetics, Physiology and Plant Protection	Governmental	0	0	0	0	20, Padurii str.	Chisinau	MD-2002	(+373 22) 77 04 47	(+373 22) 55 6180	asm_igfpp@yahoo.com	http://igfpp.asm.md/	46.98	28.89	null	11/7/2017	MDA011
FAO WIEWS	NZL001	1	NZL001	AGRESEARCH	Margot Forde Forage Germplasm Centre, AgResearch Ltd	Governmental	0	0	1	0	Grasslands Research Centre, Tennent Drive, Private Bag 11008	Palmerston North		(+64) 63518012	(063)3567399	kioumars.ghamkhar@agresearch.co.nz	http://www.agresearch.co.nz/seeds	-40.2	175.39	null		NZL001
FAO WIEWS	PAK001	10	PAK001	PGRP	Plant Genetic Resources Program	Governmental	1	0	1	1	Park Road, Islamabad	Islamabad	45500	92-51-9255203	92-51-9255201	shahid617@yahoo.com	http://www.parc.gov.pk/index.php/en/pgrp-home	33.34	73.08	null		PAK001
FAO WIEWS	PER030	2	PER030	INIA-EEA.A	Estación Experimental Agraria Andenes	Governmental	1				Av. Micaela Bastidas No. 310-314	Wanchaq, Cusco		084-232871/084-247096	084-232871	andenes@inia.gob.pe	http://www.inia.gob.pe	-13.53	-71.94	null	12/12/2014	PER030
FAO WIEWS	PER110	8	PER110	INIA-CE.LM	Centro Experimental La Molina	Governmental	1				Av. La Molina 1981	Lima 12, La Molina		01-3495955	01-3495955	cexperimlm@inia.gob.pe	http://www.inia.gob.pe	-12.01	-77.03	null	12/12/2014	PER110
FAO WIEWS	PHL180	5	PHL180	BPI-LGNCRDSC	Bureau of Plant Industry-La Granja National Crop Research, Development and Production Support Center	Governmental	1	0	1	1	La Granja	La Carlota City, Negros Occidental	6130	(034)7350088	(034)7350088	lgncrdc_lagranja@yahoo.com	http://www.bpi_lagranja@yahoo.com	10.4088	122.9832	null		PHL180
FAO WIEWS	PHL213	5	PHL213	BPI-LBNCRDPSC	Bureau of Plant Industry-Los Baños National Crop Research, Development and Production Support Center	Governmental	1	0	1	0	Economic Garden, Los Baños	Laguna	4030	(049) 536-0104	(049) 536-0104	bpi-eg 98@laguna.net		14.1755	121.2216	null		PHL213
Genesys	POL003	3	POL003	IHAR	Plant Breeding and Acclimatization Institute	Governmental	1	0	0	0	Radzików	Blonie	05-870	+48 22 725 36 11	+48 22 725 47 14	w.podyma@ihar.edu.pl	http://www.ihar.edu.pl/	52.213807	20.644048	null	12/3/2019	POL003
FAO WIEWS	POL003	3	POL003	IHAR	Plant Breeding and Acclimatization Institute	Governmental	1	0	0	0	Radzików	Blonie	05-870	+48 22 725 36 11	+48 22 725 47 14	w.podyma@ihar.edu.pl	http://www.ihar.edu.pl/	52.213807	20.644048	null	12/3/2019	POL003
Genesys	POL101	4	POL101	InHort	Research Institute of Horticulture	Governmental	1	0	1	1	Konstytucji 3 Maja 1/3 Str.	Skierniewice	96-100	+48 46 8346786, +48 46 8333434	+48 46 833 31 86	io@inhort.pl	http://www.inhort.pl/	51.960309	20.143537	null	9/1/2018	POL101
FAO WIEWS	POL101	4	POL101	InHort	Research Institute of Horticulture	Governmental	1	0	1	1	Konstytucji 3 Maja 1/3 Str.	Skierniewice	96-100	+48 46 8346786, +48 46 8333434	+48 46 833 31 86	io@inhort.pl	http://www.inhort.pl/	51.960309	20.143537	null	9/1/2018	POL101
Genesys	PRT001	154	PRT001	BPGV-DRAEDM	Portuguese Bank of Plant Germplasm	Governmental	1		1		Quinta S. José – S.Pedro de Merelim	Braga	4700-859	(+351)253300962	(+351)253300961	bpgv@draedm.min-agricultura.pt;		41.32	-8.26	null	12/12/2014	PRT001
FAO WIEWS	PRT001	154	PRT001	BPGV-DRAEDM	Portuguese Bank of Plant Germplasm	Governmental	1		1		Quinta S. José – S.Pedro de Merelim	Braga	4700-859	(+351)253300962	(+351)253300961	bpgv@draedm.min-agricultura.pt;		41.32	-8.26	null	12/12/2014	PRT001
Genesys	PRT102	1	PRT102	ISOPlexis	Banco de Germoplasma - Universidade da Madeira	Governmental	1		1	1	Campus da Penteada	Funchal - Madeira	9000-390	(+351) 291705386	(+351) 291705036	isoplexis@uma.pt	http://www.uma.pt/cem/isoplexis/	32.65911	-16.92434	null		PRT102
Genesys	ROM007	1	ROM007	BRGV Suceava	Suceava Genebank	Governmental	1	0	1	1	Bdul 1 Mai No.17	Suceava	720224	0040-230-521016	0040-230-521016	genebank@suceava.astral.ro	www.svgenebank.ro	47.55	26.32	null		ROM007
FAO WIEWS	ROM007	1	ROM007	BRGV Suceava	Suceava Genebank	Governmental	1	0	1	1	Bdul 1 Mai No.17	Suceava	720224	0040-230-521016	0040-230-521016	genebank@suceava.astral.ro	www.svgenebank.ro	47.55	26.32	null		ROM007
Genesys	SVK001	1	SVK001	SVKPIEST	Plant Production Research Center Piestany	Governmental	1				Bratislavska cesta 122	Piestany	921 68	-7721923	-7725918	benedikova@vurv.sk	http://www.cvrv.sk/en/introduction/	48.35	17.5	null	12/12/2014	SVK001

FAO WIEWS	SVK001	1	SVK001	SVKPIEST	Plant Production Research Center Piestany	Governmental	1				Bratislavska cesta 122	Piestany	921 68	-7721923	-7725918	benedikova@vurv.sk	http://www.cvrv.sk/en/introduction/	48.35	17.5	null	12/12/2014	SVK001
Genesys	SWE054	3	SWE054	NORDGEN	Nordic Genetic Resource Center	Regional	1	0	0	0	P.O. Box 41	Alnarp	SE-230 53	+46 (0)40 536640	+46 (0)40 536650	nordgen@nordgen.org	http://www.nordgen.org	55.39	13.05	null		SWE054
FAO WIEWS	SWE054	3	SWE054	NORDGEN	Nordic Genetic Resource Center	Regional	1	0	0	0	P.O. Box 41	Alnarp	SE-230 53	+46 (0)40 536640	+46 (0)40 536650	nordgen@nordgen.org	http://www.nordgen.org	55.39	13.05	null		SWE054
FAO WIEWS	SYR002	3	SYR002	ICARDA	International Centre for Agricultural Research in Dry Areas	CGIAR	1	0	0	0	P.O. Box 5466	Aleppo		(963-21)2213433	(963-21)2213490	icarda@cgiar.org	http://www.icarda.cgiar.org/	36.09	37.09	null		SYR002
FAO WIEWS	TUN029	78	TUN029	BNG	Banque national de gènes de Tunisie	Governmental	1	0	1	0	Boulevard du Leader Yasser Arafat, 1080 Charguia 1	Tunis					www.bng.nat.tn	36.844274	10.20675	null	9/1/2018	TUN029
FAO WIEWS	TUR001	1	TUR001	AARI	Plant Genetic Resources Department	Governmental	1				P.O. Box 9, Menemen	Izmir	35661	(90-232)8461331	(90-232)8461107	pgr@aari.gov.tr	http://www.aari.gov.tr	38.34	27.03	null	12/12/2014	TUR001
Genesys	TWN001	4	TWN001	AVRDC	World Vegetable Center	Non-Governmental	1	0	0	0	P.O. Box 42	Shanhua, Tainan	741	886 6 583 7801	886 6 583 0009	info@worldveg.org	http://www.avrdc.org/	23.114203	120.2982	null		TWN001
FAO WIEWS	TWN001	4	TWN001	AVRDC	World Vegetable Center	Non-Governmental	1	0	0	0	P.O. Box 42	Shanhua, Tainan	741	886 6 583 7801	886 6 583 0009	info@worldveg.org	http://www.avrdc.org/	23.114203	120.2982	null		TWN001
Genesys	UKR018	169	UKR018	IEL	Institute of Volatile Oil Bearing and Medicine Crops	Governmental					Vul. Kyivs'ka 150	Simferopol', Crimea	95034	(380652)223405/67055	(380652)223419	efirm@cris.crimea.ua; efirm@pop.cris.net		44.57	34.05	null	12/12/2014	UKR018
FAO WIEWS	UKR018	169	UKR018	IEL	Institute of Volatile Oil Bearing and Medicine Crops	Governmental					Vul. Kyivs'ka 150	Simferopol', Crimea	95034	(380652)223405/67055	(380652)223419	efirm@cris.crimea.ua; efirm@pop.cris.net		44.57	34.05	null	12/12/2014	UKR018
Genesys	UKR019	256	UKR019	ILR	Research Station of Medicinal Crops	Governmental					S. Berezotocha	Lubens'kyi r-n, Poltavs'ka obl.	37535	(38053615)20536	(38053615)20536			50.01	33	null	12/12/2014	UKR019
FAO WIEWS	UKR019	256	UKR019	ILR	Research Station of Medicinal Crops	Governmental					S. Berezotocha	Lubens'kyi r-n, Poltavs'ka obl.	37535	(38053615)20536	(38053615)20536			50.01	33	null	12/12/2014	UKR019
Genesys	UKR036	1	UKR036	NBS	Nikitskyi Botanical Gardens	Governmental	1	1			S. Nikita	Yalta, Crimea	98648	(380654)335597/30	(380654)335386	nbs1812@ukr.net		44.3	34.09	null	12/12/2014	UKR036
FAO WIEWS	UKR036	1	UKR036	NBS	Nikitskyi Botanical Gardens	Governmental	1	1			S. Nikita	Yalta, Crimea	98648	(380654)335597/30	(380654)335386	nbs1812@ukr.net		44.3	34.09	null	12/12/2014	UKR036
Genesys	UKR075	2	UKR075	IFS	Kolomyia Experimental Station	Governmental					S. Piadyky	Kolomyis'kyi r-n, Ivano-Frankivs'ka obl.	78254	(3803433)23804	(3803433)23804			48.03	25.31	null	12/12/2014	UKR075
FAO WIEWS	UKR075	2	UKR075	IFS	Kolomyia Experimental Station	Governmental					S. Piadyky	Kolomyis'kyi r-n, Ivano-Frankivs'ka obl.	78254	(3803433)23804	(3803433)23804			48.03	25.31	null	12/12/2014	UKR075
Genesys	USA022	2	USA022	W6	Western Regional Plant Introduction Station, USDA-ARS, Washington State University	Governmental	1				59 Johnson Hall,P.O.Box 646402	Pullman, WA 99164-6402		(1-509)3351502	(1-509)3356654	w6@ars-grin.gov	http://www.ars-grin.gov/ars/PacWest/Pullman/	46.46	-117.09	null	12/12/2014	USA022
FAO WIEWS	USA022	2	USA022	W6	Western Regional Plant Introduction Station, USDA-ARS, Washington State University	Governmental	1				59 Johnson Hall,P.O.Box 646402	Pullman, WA 99164-6402		(1-509)3351502	(1-509)3356654	w6@ars-grin.gov	http://www.ars-grin.gov/ars/PacWest/Pullman/	46.46	-117.09	null	12/12/2014	USA022
Genesys	USA026	539	USA026	COR	National Clonal Germplasm Repository USDA, ARS	Governmental	1				33447 Peoria Road	Corvallis, Oregon 97333-2521		(1-541)750-8712	(1-541)750-8717	corkh@ars-grin.gov	http://www.ars-grin.gov/ars/PacWest/Corvallis/ncgr/ncgr.html	44.35	-123.16	null	12/12/2014	USA026
FAO WIEWS	USA026	539	USA026	COR	National Clonal Germplasm Repository USDA, ARS	Governmental	1				33447 Peoria Road	Corvallis, Oregon 97333-2521		(1-541)750-8712	(1-541)750-8717	corkh@ars-grin.gov	http://www.ars-grin.gov/ars/PacWest/Corvallis/ncgr/ncgr.html	44.35	-123.16	null	12/12/2014	USA026
Genesys	USA151	1	USA151	NAGU	National Arboretum-Germplasm Unit, USDA/ARS	Governmental					3501 New York Ave, NE	Washington, DC 20002		(202)4754829				38.91234	-76.96992	null		USA151
FAO WIEWS	USA151	1	USA151	NAGU	National Arboretum-Germplasm Unit, USDA/ARS	Governmental					3501 New York Ave, NE	Washington, DC 20002		(202)4754829				38.91234	-76.96992	null		USA151
FAO WIEWS	ZAF062	2	ZAF062	DAFF	Genetic Resources Directorate, Department of Agriculture, Forestry and Fisheries	Governmental	0	0	0	0	Private Bag X973	Pretoria	1	+27 12 319 6366	(27-12)329-7279	dgr@nda.agric.za		-25.73	28.22	null	2/5/2018	ZAF062
